# Mild olefin formation *via* bio-inspired vitamin B_12_ photocatalysis†

**DOI:** 10.1039/d0sc05925k

**Published:** 2020-12-08

**Authors:** Radha Bam, Alexandros S. Pollatos, Austin J. Moser, Julian G. West

**Affiliations:** Department of Chemistry, Rice University 6500 Main St Houston TX USA jgwest@rice.edu .westchem.org

## Abstract

Dehydrohalogenation, or elimination of hydrogen-halide equivalents, remains one of the simplest methods for the installation of the biologically-important olefin functionality. However, this transformation often requires harsh, strongly-basic conditions, rare noble metals, or both, limiting its applicability in the synthesis of complex molecules. Nature has pursued a complementary approach in the novel vitamin B_12_-dependent photoreceptor CarH, where photolysis of a cobalt–carbon bond leads to selective olefin formation under mild, physiologically-relevant conditions. Herein we report a light-driven B_12_-based catalytic system that leverages this reactivity to convert alkyl electrophiles to olefins under incredibly mild conditions using only earth abundant elements. Further, this process exhibits a high level of regioselectivity, producing terminal olefins in moderate to excellent yield and exceptional selectivity. Finally, we are able to access a hitherto-unknown transformation, remote elimination, using two cobalt catalysts in tandem to produce subterminal olefins with excellent regioselectivity. Together, we show vitamin B_12_ to be a powerful platform for developing mild olefin-forming reactions.

## Abstract 2

Terminal or subterminal olefins can be selectively formed from alkyl electrophiles *via* bio-inspired vitamin B_12_ photocatalysis.

## Graphical abstract

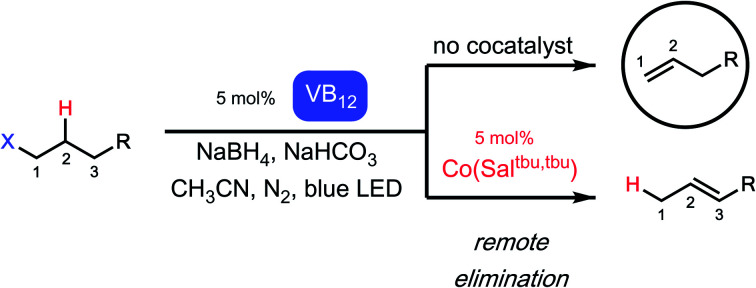

## Introduction

Vitamin B_12_ (VB_12_), or cobalamin, is one of nature's most versatile and intriguing cofactors.^1–5^ Aside from its unparalleled structural complexity and exceptional size, it is notable for the breadth of chemical reactions it catalyzes, from methyl transferase activity in the biosynthesis of methionine to 1,2-transposition of substituents in isomerase enzymes. The manner by which VB_12_ accesses this diverse reactivity is also highly unique: its catalytic cycles involve direct association of organic substrates to the central cobalt atom of the cofactor, generating discrete organometallic intermediates. These intermediates can then be engaged in either ionic, 2 electron elementary steps or radical, 1 electron steps depending on the enzymatic microenvironment, permitting tunable reactivity from a common cofactor and allowing for powerful chemical transformations to be achieved.^3,4,6^

**Fig. 1:**
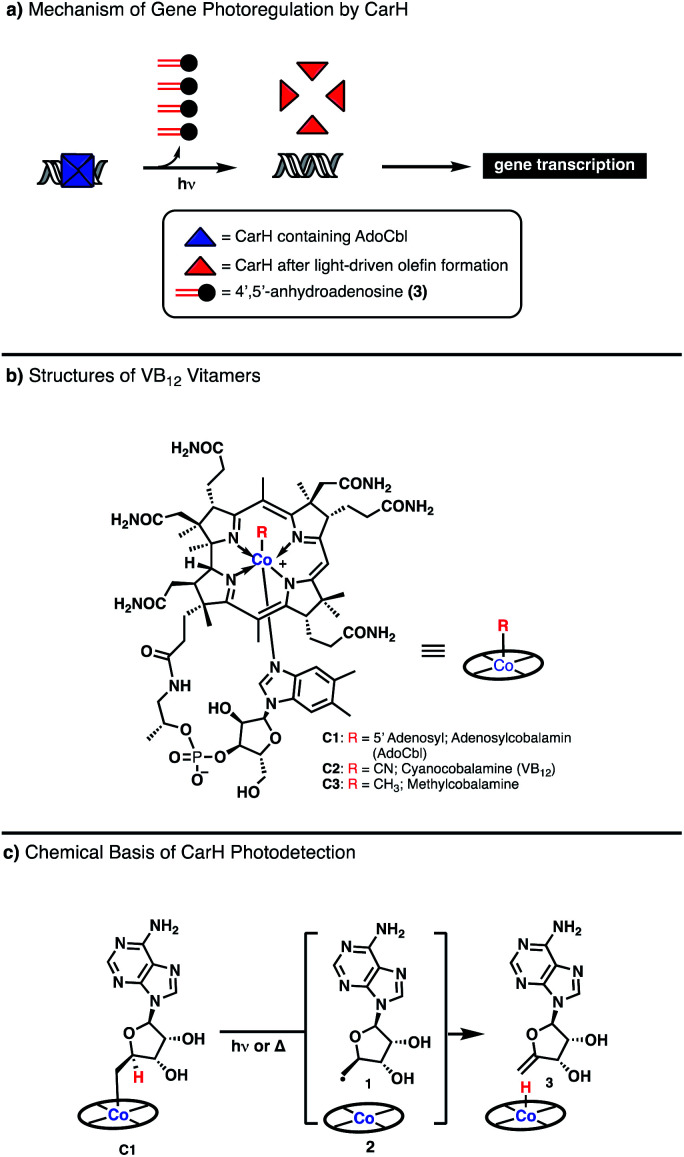
The mechanism of photodetection by VB_12_ in the enzyme CarH.

The vast majority of VB_12_-catalyzed reactions in biology occur in a light-independent fashion, with the chemical steps driven thermally.^4^ However, recent investigation of the photoreceptor CarH from *T. thermophilus*, has demonstrated a completely new reaction modality for VB_12_ driven by visible light (Fig. 1, Panel A).^7^ CarH exists as a tetramer binding the promoter region of light-controlled genes, with each copy containing the VB_12_ derivative adenosyl cobalamin (AdoCbl, C1). Upon irradiation, the cobalt–carbon bond of AdoCbl fragments, leading to conformational change and dissociation of the CarH tetramer, releasing the bound DNA to be transcribed. Most interestingly, the light-driven cleavage of the cobalt–carbon bond does not release the expected product, the highly-reactive adenosyl radical but, rather, the much more inert 4′,5′-anhydroadenosine, an unprecedented enzymatic olefin-forming transformation for VB_12_ (Fig. 1, Panels A and C).^8^

Toward understanding this unusual observation, Drennan and coworkers interrogated the mechanism of this process, building on the observations of Finke who studied the homolytic cleavage of the Co–C bond of adenosylcobalamin (AdoCbl) C1 molecule, a relatively weak bond (∼31 kcal mol^−1^), to generate a adenosyl radical (Ado˙) 2 and a Co(ii) metalloradical (Fig. 1b).^9,10^ If no competitive radical trap is present, it was found that the Co(ii) can abstract a hydrogen atom from the position adjacent the carbon-centered radical to afford a terminal olefin stoichiometrically (Fig. 1, Panel C). This *in vitro* reactivity was found to be the key step in the *in vivo* photodetection of CarH, a completely new function of VB_12_ in biology.^7,8^

As synthetic chemists, we were immediately drawn to this unprecedented reactivity of vitamin B_12_ as a potential means of synthesizing olefins. Olefins are an important functional group found in many molecules important for modern life, from pharmaceuticals to next generation materials.^11–13^ Further, olefins are exceptionally-useful as functionalization sites during synthesis.^14–16^ Traditionally in organic synthesis, access to olefins has been limited to the olefination of carbonyls or elimination of HX (X = halides, sulfonates, OH, OR, *etc.*) from a suitably-prefunctionalized alkane. Unfortunately, such eliminations often demonstrate a profound lack of stereo- and regioselectivity.^17,18^ These issues are further exacerbated by the use of strong base, which imparts both a lack of chemoselectivity and functional group tolerance. Importantly, elimination methods often favor the formation of the thermodynamically-stable internal alkene over terminal alkenes, an observation known as Zaitsev's rule.^19^

**Fig. 2:**
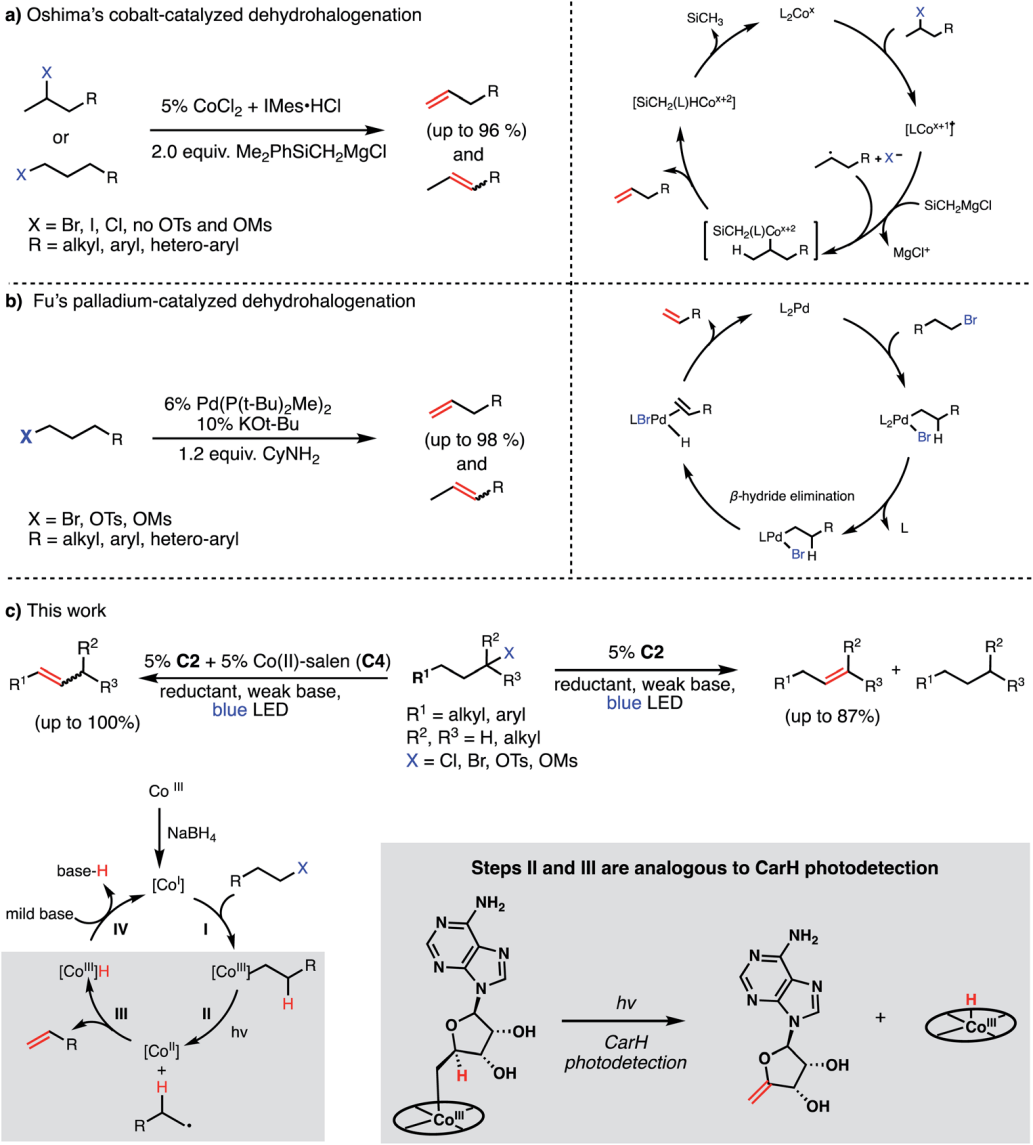
Catalytic methods for olefin formation from alkyl electrophiles and their proposed mechanisms. (a) Cobalt-catalyzed dehydrohalogenation developed by Oshima, (b) palladium-catalyzed dehydrohalogenation developed by Fu, (c) this work.

Recent efforts in organometallic chemistry have delivered some promising alternatives to traditional elimination reactions. In 2008, Oshima reported the use of CoCl_2_/IMes·HCl in conjunction with 2 equiv. of a Grignard reagent (Me_2_PhSiCH_2_MgCl) to convert 2-bromoalkanes (and some 1-bromoalkanes) to terminal olefins in good to excellent yields (79–96%, Fig. 2, Panel A).^17^ The method was not found to be effective for producing olefins from chlorides (poor yields) or alkyl sulfonates (due to the high barrier for C–O bond homolysis). Further, the use of a Grignard reagent presents similar issues of functional group intolerance and chemoselectivity as seen in traditional strong base-mediated eliminations. Four years later, the Fu group reported a Pd(P(*t*-Bu)_2_Me)_2_-catalyzed dehydrohalogenation of primary alkyl bromides and sulfonates to give terminal alkenes in good yields (84–99%, Fig. 2, Panel B).^18^ While avoiding the stoichiometric Grignard reagents of Oshima's system and delivering olefins with high efficiency, Fu's method similarly presents potential issues in chemoselectivity by using strong base (KO*t*-Bu or LiOMe) and performs poorly when using alkyl chlorides, likely due to slow oxidative addition to the palladium catalyst. Finally, this method relies on palladium as a catalyst, an exceptionally-rare and expensive element.

While the development of methods for dehydrohalogenation to give olefins (in particular, terminal olefins) has made significant headway, a truly mild and sustainable method remains elusive in organic synthesis. Toward addressing this gap, we sought to adapt the photochemistry of CarH into a synthetic method, rendering the olefin formation catalytic and allowing for various alkyl electrophiles to be engaged in this desirable transformation.

We envisioned our putative catalytic cycle starting from the Co(i) state of VB_12_, a “supernucleophile” that is known to readily alkylate with many alkyl electrophiles, generating a Co(iii) alkyl intermediate (Fig. 2, Panel C).^6,20^ In particular, this Co(i) “supernucleophile” is relatively insensitive to steric hindrance in the S_N_2 transition state, potentially allowing for a large substrate scope.^20^ Following this, we imagined that this Co(iii) alkyl might be photolyzed to produce a Co(ii) metalloradical and alkyl radical which can then collapse to produce the desired alkene and a cobalt hydride, a sequence analogous to the photodetection reaction of CarH.^7,8^ Finally, deprotonation of this highly-acidic hydride by weak base would regenerate the Co(i) supernucleophile, closing the catalytic cycle. With regard to precatalyst, we envisioned that we might be able to reduce an air-stable Co(iii) precursor (*e.g.* cyanocobalamin) *in situ* using a mild reductant such as sodium borohydride, a known approach to generating the supernucleophilic Co(i) state.^6,20^ Together, this cycle would allow for the mild dehydrohalogenation of alkyl electrophiles using weak base and visible light energy.

## Results and discussion

### Initial reaction development

We set out to explore the possibility of a VB_12_ photocatalyzed dehydrohalogenation reaction using the simple alkyl halide 1-chlorooctane (Table 1, 2a) as a model substrate. To our delight, when alkyl chloride 2a was treated with a catalytic amount of VB_12_C2 (5 mol%), 4.0 equivalent of NaBH_4_, and 1.5 equiv. NaHCO_3_ at room temperature for 16 h under blue LED light (*λ*_max_ = 427 nm), the expected dehydrohalogenation of the substrate proceeded smoothly to give terminal alkene (3a) in 75% yield (Table 1, entry 1). We next endeavored to explore the necessary elements of the reaction to gain some inkling as to whether our mechanistic design was valid. First, we established that visible light is required, as no olefin was formed in the absence of blue light (Table 1, entry 2). Further, we confirmed the necessity of both VB_12_ (C2) and mild base, NaHCO_3_ (Table 1, entry 3), with the starting material being recovered unchanged in the absence of either.

**Table 1:** Development of VB_12_-photocatalyzed olefination of alkyl halides

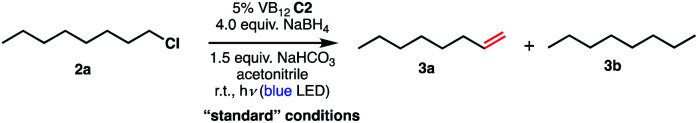			
Entry	Variation from optimized conditions	Time	% yield 3a^a^ (% conversion)
1	None	16 h	75 (100^b^)
2	No light	2 d	0 (0^c^)
3	No C2 or no NaHCO_3_	2 d	0 (0^c^)
4	Phenylsilane or B_2_Pin_2_ instead of NaBH_4_	2 d	0 (0^c^)
5	Zn or Mn instead of NaBH_4_	22 h	0 (0^c^)
6	Triethylamine instead of NaHCO_3_	2 d	0 (0^c^)
7	K_2_CO_3_ instead of NaHCO_3_	20 h	35 (100^b^)
8	1.0 equiv. Na_2_CO_3_ instead of 1.5 equiv. NaHCO_3_	18 h	36 (50^b^)
9	1.5 equiv. NaBH_4_	16 h	56 (100^b^)
10	5% COPC instead of 5% C2	21 h	0 (100^d^)
11	5% AdoCbl (C1) instead of 5% C2	15 h	34 (100^b^)
12	DMF instead of acetonitrile	16 h	35 (100^b^)
13	DMSO instead of acetonitrile	24 h	17 (100^b^)
14	Acetone instead of acetonitrile	24 h	21 (100^b^)

a Light irradiation [LED, *λ*_max_ = 427 nm]. Determination of yields *via* NMR using 1,3,5-trimethoxybenzene as an internal standard. All reactions were carried out using 2a applying standard conditions (0.1 mmol) in 0.1 M concentration under a N_2_ atmosphere at room temperature.

b Remaining mass balanced by side product 3b.

c Only starting material.

d ∼40% internal alkene and 3b side product.

Attempts to replace various components of this reaction revealed this combination to be uniquely privileged for olefin formation. Indeed, efforts to substitute sodium borohydride (NaBH_4_) with other organic reductants such as phenylsilane or B_2_Pin_2_ were unsuccessful (Table 1, entry 4), as were those using the inorganic reductants zinc (Zn) or manganese (Mn) (Table 1, entry 5). Similarly, no productive reaction occurred when our weak inorganic base NaHCO_3_ was substituted by equal amount of triethylamine (Table 1 entry 6). Other inorganic bases such as Na_2_CO_3_ and K_2_CO_3_, while successful in olefination, functioned significantly less efficiently (Table 1, entries 7 and 8) Interestingly, when the amount of NaBH_4_ was lowered from 4.0 equivalents to 1.5 equivalents, the efficiency of the reaction was significantly reduced, only producing 56% of the desired product (Table 1, entry 9). While stoichiometric reductant is not required in principle, the exceptional sensitivity of Co(i) and Co(ii) to adventitious oxidants might necessitate high quantities to rescue any oxidized cobalt.^10^ Additionally, the Co(iii)–H state is known to be capable of hydrogen evolution, presenting a parasitic oxidation pathway of this key intermediate that would require reductant to return to the Co(i) supernucleophile.^21^ We also wondered whether related cobalt complexes might be competent for the reaction. Gratifyingly, we found that the vitamer AdoCbl, the active species in CarH photodetection, is a suitable precatalyst, though its efficiency is significantly lower than that of C2. By contrast, VB_12_ mimic compound cobaloxime pyridine chloride (COPC) was ineffective for the reaction, suggesting the true corrin environment of VB_12_ is essential for desired reactivity (Table 1, entries 10 and 11).^22^ This result is of particular interest in light of the prevalence of cobaloximes in the HAT literature.^11,23–28^ Finally, we found acetonitrile to be the most selective solvent for this process. Low selectivity was observed on using dimethylforamide (DMF), with the yield of desired terminal alkene 3a decreasing to 35% and remainder of the mass balance corresponding to side product 3b (Table 1 entry 12). Similarly, the catalytic action of our method was greatly impacted by the polar solvents dimethyl sulfoxide (DMSO) and acetone, favoring the formation of reduced side product 3b, with olefinic product 3a being formed in 17% and 21% respectively (Table 1, entries 13 and 14).

### Dehydrohalogenation of alkyl halides to form terminal olefins

With an efficient catalytic method in hand, we next sought to explore the substrate scope of our mild VB_12_-catalyzed dehydrohalogenation method and found a variety of alkyl halide substrates to function effectively (Table 2).

**Table 2:** VB_12_-catalyzed olefination reaction of alkyl halides

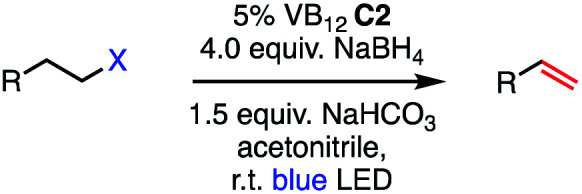			
Entry	Substrate	Product	% yield^a^ (% conversion)
1	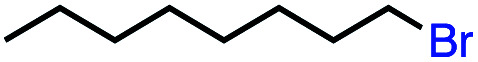	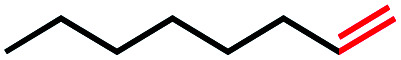	82 (100^b^)
2	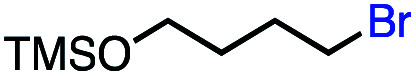	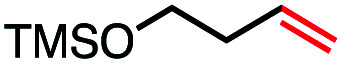	60 (100^b^)
3	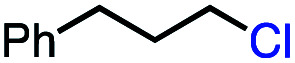	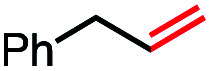	74 (100^b^)
4	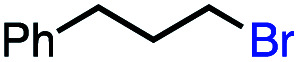	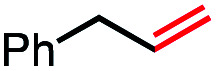	75 (100^b^)
5	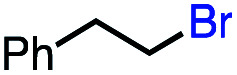	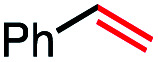	50 (100^b^)
6	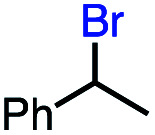	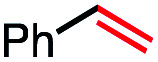	25 (100^b^)
7	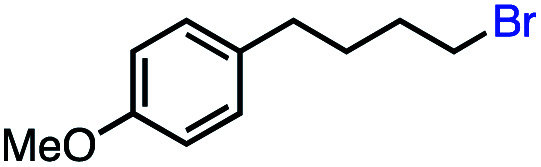	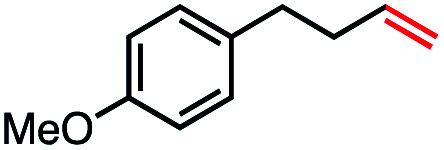	71 (100^b^)
8	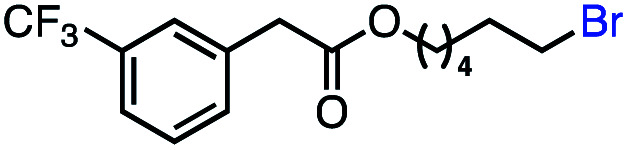	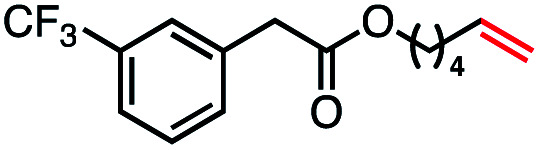	57 (100^b^)
9	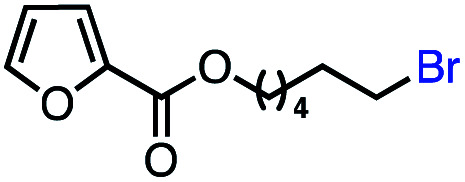	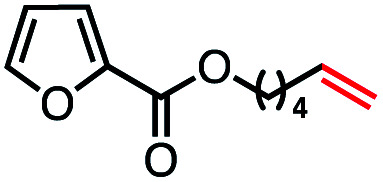	61 (100^b^)
10	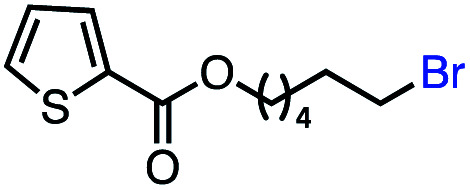	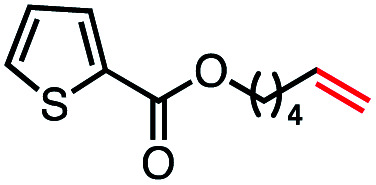	66 (100^b^)
11	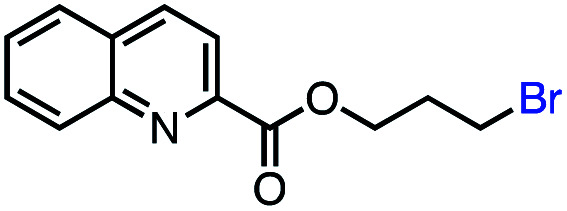	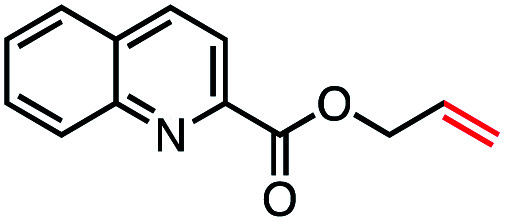	53 (85^b^)
12	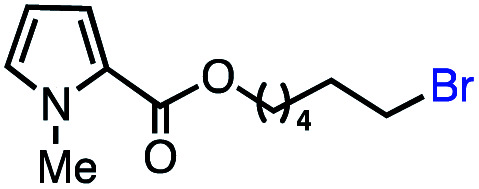	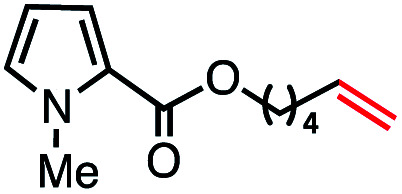	69 (100^b^)
13	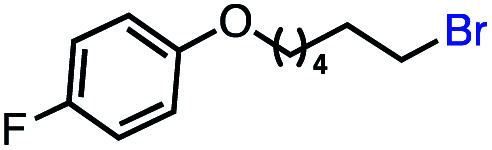	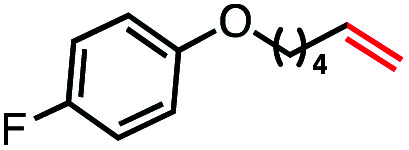	55 (100^b^)
14	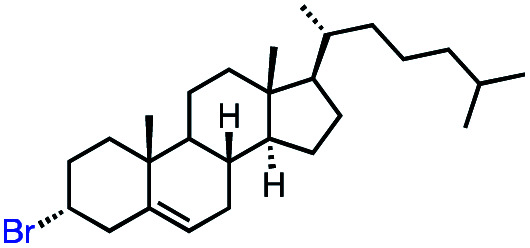	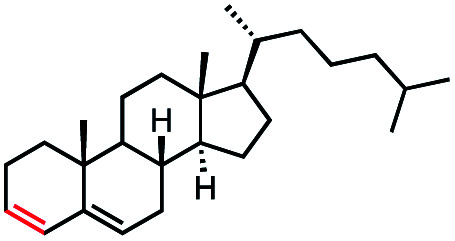	52 (70^b^^,^^c^)
15	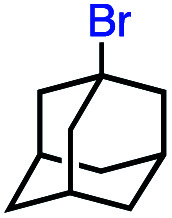	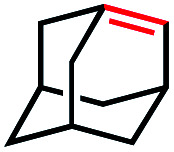	0 (0)

a Light irradiation [LED, *λ*_max_ = 427 nm]. Determination of yields *via* NMR using 1,3,5-trimethoxybenzene as an internal standard. All reactions were carried out applying standard conditions (0.1 mmol) substrate in 0.1 M concentration under a N_2_ atmosphere at room temperature.

b Remaining mass balance is reduced side product.

c Used THF/DMF(1 : 1 ratio, v/v) as solvent and 10 mol% VB_12_, reaction time *ca.* 23 h.

In our initial investigation on the scope of our catalytic method, we found that unactivated primary alkyl halides undergo the dehydrohalogenation reaction smoothly, providing desired products in good yields (82% and 60% respectively; Table 2, entry 1 and 2). Importantly, TMS ether functionality is preserved under our mild reaction conditions, in contrast to its instability under strongly-basic Wittig conditions.^29^ Aromatic substitution of the substrate is also well tolerated by our reaction conditions, with 3-phenylpropyl chloride, 3-phenylpropyl bromide and 2-phenylethyl bromide furnishing the desired terminal products in 74%, 75% and 50% respectively (Table 2, entries 3–5). Reaction of 1-bromoethyl benzene proceeds to completion; however, the yield of the desired terminal alkene is low, with the major product being ethylbenzene, the protodehalogenation product (Table 2, entry 6). A variety of electron rich and poor aromatics function well in this method, as do oxygen, sulfur, and nitrogen-containing heterocycles (Table 2, entries 7–13). We established that this method can function on biologically-relevant substrates, dehydrohalogenating bromocholesterol to form cholesta-3,5-diene (Table 2, entry 14). Finally, it was shown that 1-bromoadamantane is completely unreactive under our conditions, an expected result both due to its inability to participate in S_N_2 alkylation of the Co(i) supernucleophile and the instability of the hypothetical anti-Bredt olefin product (Table 2, entry 15).^30^

Based on this success, we questioned whether other alkyl electrophiles might be suitable for our reaction conditions, particularly alkyl sulfonates. To our delight, we found this to be the case, with both cyclic and acyclic tosylates leading to olefins in good to moderate yields (Table 3, entries 1–5). Interestingly, when we subjected a secondary tosylate to our reaction conditions, we found strong preference for the terminal isomer, demonstrating a high level of regioselectivity in our method (Table 3, entry 4). Mesylates were also well-behaved in our system, forming terminal olefins in good to excellent yields (Table 3, entry 6 and 7).

**Table 3:** VB_12_-catalyzed olefination reaction of alkyl sulfonates

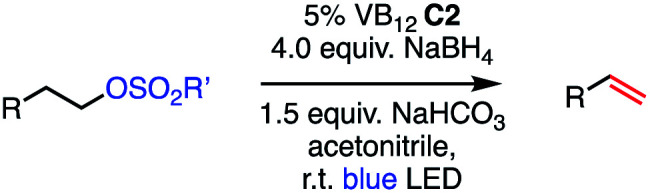			
Entry	Substrate	Product	% yield^a^ (% conversion)
1	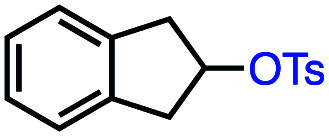	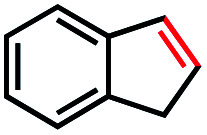	87 (100^b^)
2	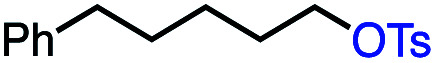	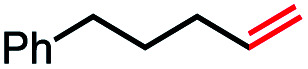	54 (98^b^)
3	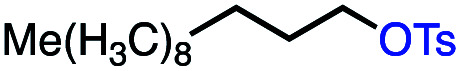	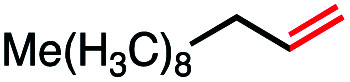	56 (100^b^)
4	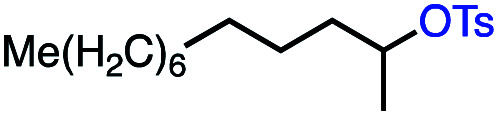	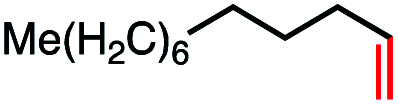	56 (76^c^)
5	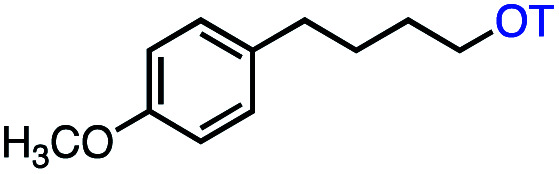	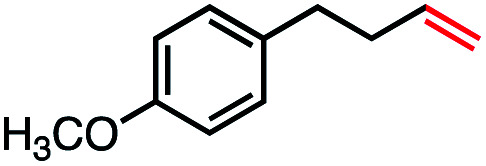	75 (70^b^)
6	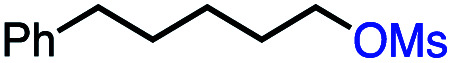	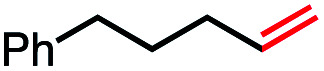	88 (100^b^)
7	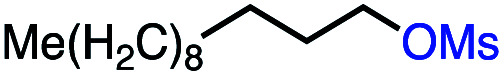	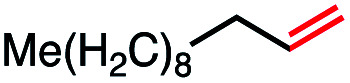	53 (78^b^)

a Light irradiation [LED, *λ*_max_ = 427 nm]. Determination of yields *via* NMR using 1,3,5-trimethoxybenzene as an internal standard. All reactions were carried out applying standard conditions (0.1 mmol) substrate in 0.1 M concentration under N_2_ atmosphere at room temperature.

b Remaining mass balance is reduced side product.

c 20% internal alkene.

### Catalytic remote eliminations

As we established the considerable generality of this bio-inspired olefination, we found ourselves wondering if we might build on its unique characteristics, notably the intermediacy of photo-triggered radical species, to design hitherto unknown reactions. Indeed, our initial reaction design leverages the ability of the VB_12_ Co(ii) metalloradical to act on alkyl radical intermediates, perpetrating a hydrogen atom transfer (HAT) to generate terminal olefin products and a Co(iii) hydride (Co(iii)–H) intermediate. This cobalt hydride intermediate immediately caught our interest, as an analogous Co(iii)–H generated from the cobalt salen complex C4 had recently been shown by Shenvi and coworkers to regioselectivity isomerize terminal olefins, forming the subterminal olefins with exquisite selectivity.^31^

A recent cobalt system developed by Findlater and coworkers has similarly been efficient for terminal olefin isomerization and mechanistic study has implicated cobalt hydride intermediates.^32^ Iron complexes have recently been reported to catalyse a similar transformation.^33^

Taking these two observations together, we envisioned a completely new reaction, 2,3-elimination (Table 4). As our reaction selectively forms terminal olefins and the C4 system isomerizes terminal olefins selectively, we posited that it might be possible to perform a tandem olefination/isomerization reaction to generate subterminal olefins from primary alkyl halides. This “remote elimination” process would replace a primary alkyl electrophile with hydrogen while also installing an olefin adjacent to the original site of the leaving group, functioning as a regioselective halide-directed C–H desaturation reaction, a completely unprecedented transformation and complementary approach to remote functionalization proceeding *via* HAT to carbon-centered radicals.^34–39^

**Table 4:** Remote elimination of alkyl substrates *via* dual cobalt catalysis

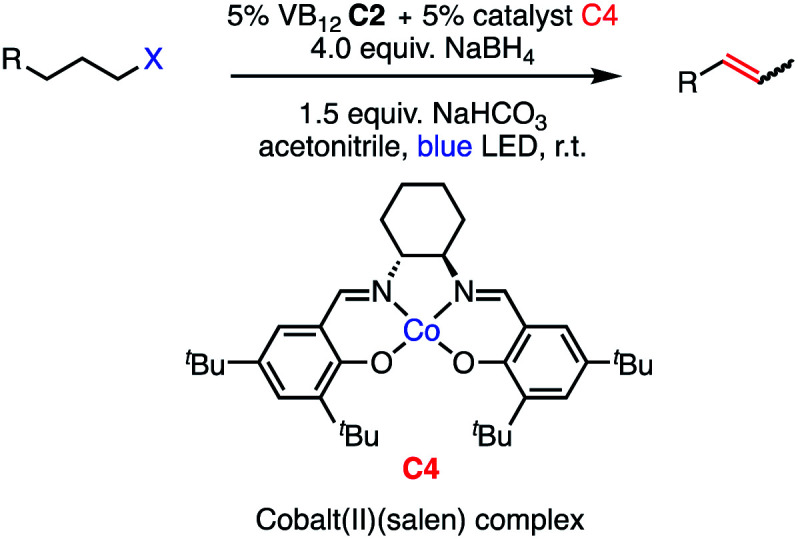				
Entry	Substrate	Product	% yield^a^	% conversion (*E*/*Z* ratio)
1	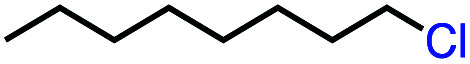	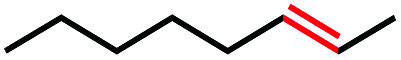	100	100^b^ (3 : 1)
2	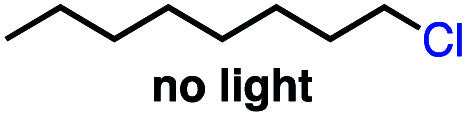	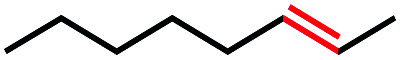	0	0
3	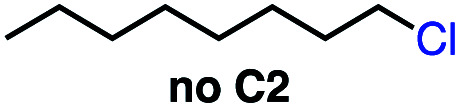	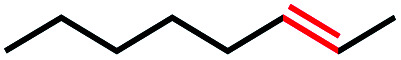	0	0
4	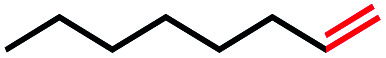	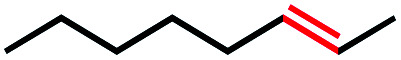	4	4^c^ (N/A)
5	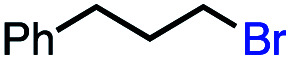	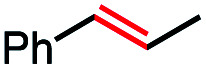	40	100^d^ (>10 : 1)
6	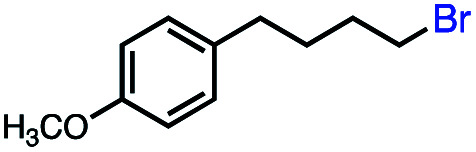	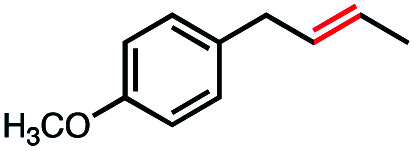	41	100^e^ (3 : 1)
7	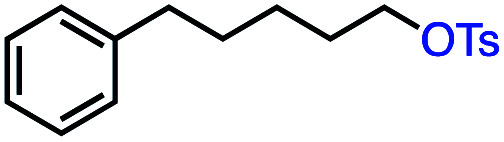	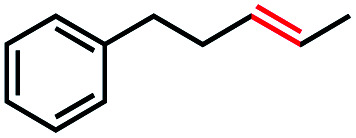	52	100^f^ (2 : 1)
8	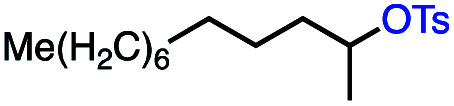	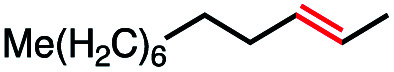	66	N/A^f^ (3 : 1)

a Light irradiation [LED, *λ*_max_ = 427 nm]. Determination of yields *via* NMR using 1,3,5-tri-methoxybenzene as an internal standard. All reactions were carried out applying standard conditions with (0.1 mmol) substrate in 0.1 M concentration under N_2_ atm at room temp.

b Reaction NMR after 18 h.

c Reaction checked after 20 h.

d Remaining mass balance is reduced side product (47%) along with terminal alkene (13%).

e Remaining mass balance is reduced side product.

f Remaining mass balance is an unresolvable mixture of reduced side product, terminal alkene, and starting material.

Seeking to test this hypothesis, we subjected 1-chlorooctane to our optimized olefination conditions with 5 mol% of cobalt complex C4 added. Gratifyingly, we obtained the internal alkene selectively with quantitative yield under these conditions (Table 3, entry 1). Subsequent control reactions established that both light and VB_12_ complex C2 are required for this reaction, with no 2-alkene being formed in the absence of either (Table 3, entries 2 and 3). We also wondered whether this system would be competent for regioselective olefin isomerization in analogy to the observations of Shenvi.^31^ To our surprise, subjecting 1-octene to our remote elimination conditions led to minimal isomerization to the subterminal olefin 2-octene (Table 3, entry 4). Taking all these observations together, it appears that the presence of both cobalt catalysts and light are essential for remote elimination of alkyl electrophiles.

With these optimized conditions in hand, we next sought to test the substrate tolerance of this novel reaction. In addition to alkyl chlorides, we were pleased to find that alkyl bromides function with moderate to good efficiency in the reaction (Table 4, entries 5 and 6) as did alkyl tosylates (Table 4, entries 7 and 8). Interestingly, we found the regioselectivity of secondary alkyl electrophile capable of forming a terminal or subterminal olefin to be completely flipped, forming the subterminal olefin selectively under the dual-catalytic conditions (Table 4, entry 8) as opposed to the terminal olefin observed under VB_12_-only conditions (Table 3, entry 4). Further, we found the olefin isomerization to be limited to the subterminal position, with no further chain walking in cases with a thermodynamic sink such as styrene formation (Table 4, entries 6 and 7).

### Preliminary mechanistic studies

**Fig. 3:**
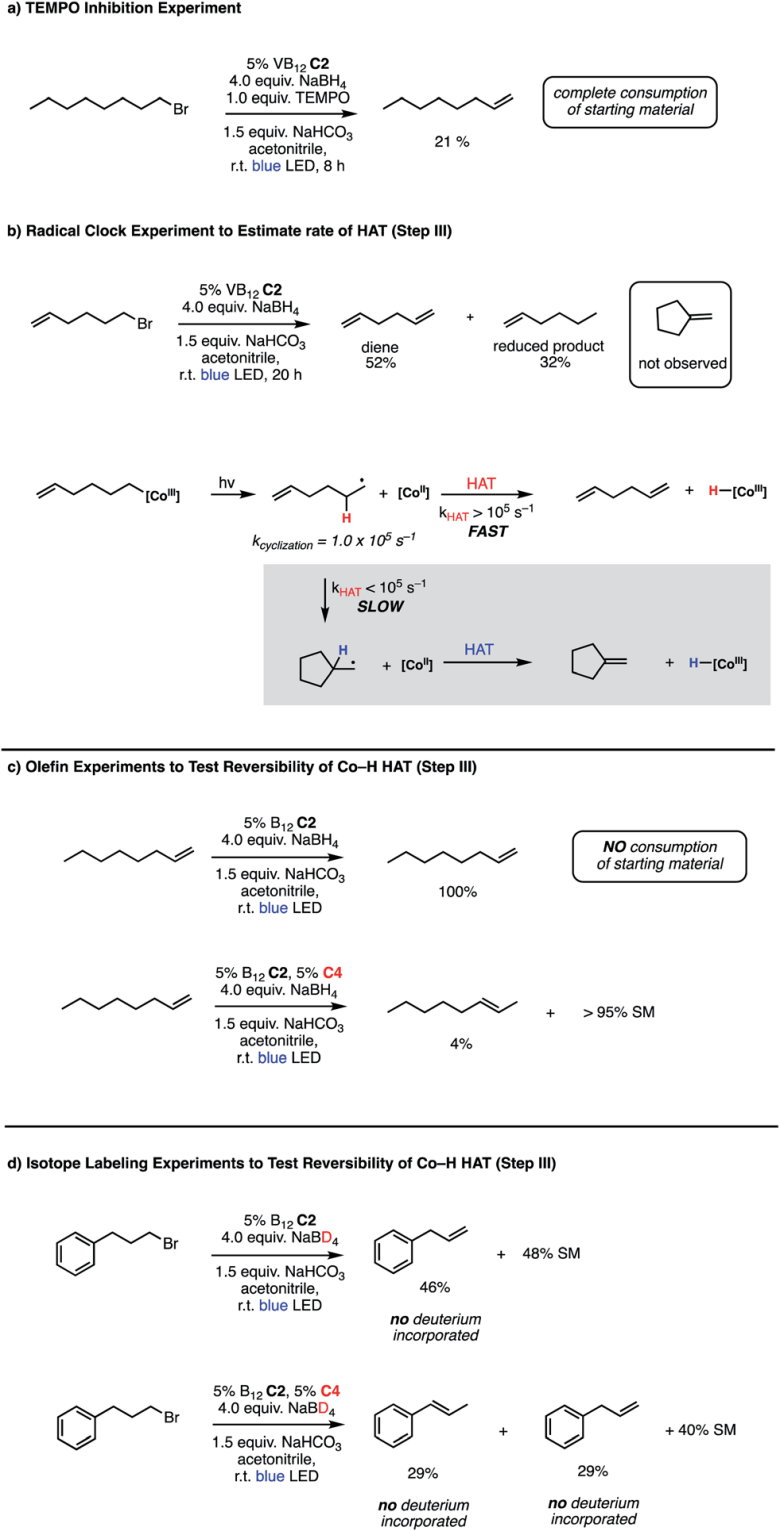
Mechanistic experiments for VB_12_-catalyzed olefin formations. (a) TEMPO inhibition, (b) radical clock, (c) olefin reactivity, (d) isotope labeling.

Bolstered by the wide scope of our photocatalytic olefination reactions, we wondered whether we could gain some preliminary insight into their mechanisms to determine whether our initial design (Fig. 2, Panel C) reflects some aspects of reality. Our first course of action was to explore the intermediacy of radical species, a key aspect of our mechanistic design. The need for photolytic conditions for the reaction to proceed was itself highly suggestive in light of the reactivity of VB_12_ alkyl products;^5,7,8,10^ however, we sought to further support this proposal through TEMPO addition experiments. Gratifyingly, inclusion of stoichiometric TEMPO results in a major reduction of reaction efficiency (Fig. 3, Panel A), an expected result for a radical transformation, though TEMPO results must be interpreted with care.^40^

With this TEMPO result in hand, we next sought to explore the lifetime of our proposed radical intermediates arising from step II of our mechanism, cobalt–carbon bond homolysis (Fig. 2, Panel C), before olefination. To test this, we used a radical clock substrate, settling on the 5-hexenyl radical as our rearrangement substrate, a clock first studied by Ingold whose rate of closure is intermediate at 1.0 × 10^5^ s^−1^ (Fig. 3, Panel B).^41,42^ Thus, if the rate of HAT to the Co(ii) metalloradical is on the order of 10^5^ s^−1^ or slower, we would expect to see significant methylenecyclopentane formation. However, if the rate of HAT is significantly faster than the rate of closure, we anticipate that the primary product of dehydrohalogenation will be 1,5-hexadiene. Subjecting 1-bromo-5-hexene to our reaction conditions led to formation of 1,5-hexadiene as the major product as well as the reduced side product 1-hexene. The complete absence of the cyclized product suggests that the Co(ii)-perpetrated HAT is quite rapid, likely proceeding at rates above 10^5^ s^−1^. While an ionic mechanism of desaturation would also be consistent with this observation, the large impact of TEMPO on our reaction in addition to the need for light irradiation leads us to favor a fast, radical HAT mechanism.

We also considered the formation of the reduced alkane product, a minor side product in some reactions, though a significant product in the case of the radical clock investigation. Oshima^43^ and Branchaud^44,45^ observed competitive alkane formation in reactions leading to olefination *via* radical cobalt mechanisms, and that this formation was independent of trace O_2_ and other impurities in solvents, suggesting that the reduction may be intrinsic to organocobalt chemistry. Importantly, this product was observed in the absence of an external hydride source, such as sodium borohydride. We found the formation of alkane side product was exacerbated by more polar solvents (Table 1, entries 10–12), potentially suggesting that this side reaction proceeds through a more polar transition state. Alkane side product formation also seems influenced by alkyl-radical stability and thus lifetime, which could allow for additional side reactions upon cage escape. This effect is demonstrated most clearly for 1-bromo-1-phenylethane, where 75% of the starting material is reduced to ethylbenzene and only 25% converted to styrene (Table 2, entry 6).

Having preliminary evidence that this cobalt-mediated HAT step is rapid, we next questioned whether it is reversible under our reaction conditions. To test this, 1-octene, a prototypical product of the olefination, was subjected to the reaction conditions (Fig. 3, Panel C), resulting in quantitative recovery of the starting material. This observation suggests that the olefin-forming step III in this reaction is irreversible and the reduced alkane side product does not arise from olefin hydrogenation. Similarly, we subjected this same starting material to the remote elimination conditions and observed 4% subterminal olefin product in 22 h, with the remainder of the mass balance being recovered starting material. We found this low isomerization activity interesting, as we expected that the system should behave similarly to that of Shenvi in the presence of olefins, especially as borohydride reductants have been shown to generate cobalt hydride species in Co(Salen) frameworks.^46^

To complement these olefin studies, we also pursued isotope labeling experiments to investigate the possibility that the olefin formation is reversible, yet simply returns starting material (step III in the proposed mechanism (Fig. 2, Panel C)). By replacing sodium borohydride with its deuterated congener, sodium borodeuteride, we reasoned that some amount of cobalt deuteride should be formed in the reaction and, if HAT is reversible, this might exchange into the terminal position of the olefin. When put into practice with 1-bromo-3-phenylpropane, we found no deuterium incorporation in the final olefin product 3-phenylpropene (Fig. 3, Panel D). Interestingly, the rate of reaction is slower using the borodeuteride reductant, with approximately half of the starting material remaining unconsumed overnight. Similarly, we saw no deuterium incorporation in the products arising from the remote elimination system in addition to incomplete consumption of starting material. An interesting further divergence of this result from the borohydride reaction is the product distribution, wherein we observe a 1 : 1 ratio of terminal and subterminal olefins, as opposed to the high selectivity for subterminal, remote elimination product found before.

The cause of this change in product selectivity for the remote elimination is not obvious. Shenvi's group performed a number of mechanistic studies to illuminate the catalytic cycle of their olefin isomerization using catalyst C4 and found strong evidence that it proceeds *via* metal-catalyzed HAT (mHAT) reactions. First, a transfer from a Co(iii)–H to the olefin generates a secondary carbon-centered radical, an intermediate which can then transfer a hydrogen atom from an adjacent carbon to form the subterminal olefin and regenerate the Co(iii)–H species.^31^ While it is likely that the function of this complex in our remote elimination system is similar, especially in light of the high selectivity for subterminal olefin formation under normal borohydride conditions, the reduction in selectivity under borodeuteride conditions suggests that the efficiency of the isomerization reaction is closely tied to the function of the VB_12_ elimination system, with a reduction in VB_12_ efficiency impacting the isomerization as well. Additionally, the lack of deuterium incorporation in isomerized product suggests that the hydrogen atom transferred by catalyst C4 arises from substrate. A substrate hydrogen might arrive at C4*via* direct trapping of the alkyl radical following photolysis of the Co–C bond of C2 (step II), Co–Co transmetalation from C2 to C4 followed by homolysis/isomerization, or potentially HAT from the C2 hydride formed after photolysis/HAT to C4 (step III). While each of these mechanisms could potentially explain this observation, the direct trapping would need to be exceptionally rapid given our radical clock results and the Co–Co HAT would be expected to permit significant deuterium crossover, a proposal inconsistent with our data.

## Conclusions

In summary, we have developed a mild, vitamin B_12_-catalyzed system for olefin formation from alkyl halides and sulfonates based on the photoactivity of the enzyme CarH. This light-driven system has exquisite regioselectivity for terminal olefin products, providing a robust method for forming these valuable products. We have also developed a dual-catalyst system utilizing a second, Co–salen catalyst in combination with VB_12_ which allows for the reaction of terminal leaving groups to form subterminal olefins capped by a methyl group, an unprecedented ‘remote elimination’ process which is a valuable addition to the synthetic chemists' toolbox. Preliminary study supports our initial mechanistic proposal for this olefin formation and suggests that the key hydrogen atom transfer step leading to olefin formation is highly efficient and irreversible under our conditions. We are excited by the possibilities afforded by this reactivity and efforts to expand its uses are ongoing in our laboratory.

## Conflicts of interest

There are no conflicts to declare.

## Supplementary Material

SC-012-D0SC05925K-s001
