# Supplementary material for: Mild olefin formation via bio-inspired vitamin B12 photocatalysis
Source: Chem Sci. 2020 Dec 8;12(5):1736–44. doi: 10.1039/d0sc05925k (PMC8179286; doi:10.1039/d0sc05925k)
Supplement: SC-012-D0SC05925K-s001 [file SC-012-D0SC05925K-s001.pdf]

Electronic Supplementary Material (ESI) for Chemical Science.
This journal is © The Royal Society of Chemistry 2020

# Mild olefin formation via bio-inspired vitamin $B_{12}$ photocatalysis

Radha Bam, Alexandros S. Pollatos, Austin J. Moser and Julian G. West*

Department of chemistry, Rice University, 6100 Main St, Houston, TX 77005
*Email: jgwest@rice.edu

## Supporting Information

## Table of Contents

**1. General Experimental Section** .......... **2**

**2. Preparation of Substrates** .......... **3**

*A. Preparation of Alkyl Bromides* .......... *3*

*B. Preparation of Alkyl Tosylates* .......... *6*

*C. Preparation of Alkyl Mesylates* .......... *8*

**3. Optimization of $VB_{12}$-Photocatalyzed Olefination of Alkyl halide** .......... **9**

**4. General Method of $B_{12}$ Catalyzed Dehydrogenation** .......... **11**

**5. General Method of Remote Elimination** .......... **11**

**6. Mechanistic Studies** .......... **18**

*A. Inhibition Experiments* .......... *18*

*B. Radical Clock Experiment* .......... *20*

*C. Isotope Labeling Experiment* .......... *20*

**$^{1}H$ and $^{13}C$ NMR Spectra of New Compounds** .......... **22**

**References** .......... **26**

## 1. General Experimental Section

The commercially available reagents were purchased at highest commercial quality and directly used without further purification. All the air or moisture-sensitive reactions were set-up using oven-dried glassware under an inert atmosphere. The reactions were monitored by thin-layer chromatography (TLC) carried out on Merck silica gel pre-coated (60 F254) glass plates (0.25mm). Purification of reaction products were carried out by flash chromatography using silica gel 60 (230-400 mesh). The NMR yields of the compounds were calculated using 1,3,5-trimethoxybenzene (proton signal at δ 6.08 ppm) as an internal standard. $^{1}H$ and $^{13}C$ NMR spectra were recorded on Bruker 600 (600 MHz). The spectra are referenced relative to residual $CD_3CN$ or $CDCl_3$ proton signals at δ 1.94 ppm and δ 7.26 ppm, respectively. The chemical shifts are reported in part per million (ppm) from high to low frequency and referred to the residual solvent resonance peak. Coupling constant (*J*) are reported in Hz. The multiplicity of $^{1}H$ signals are indicated as: s = singlet, d = doublet, t = triplet, p = pentet, m = multiplet. IR data were recorded with Bruker Alfa Platinum ATR single reflector spectrometer by applying the compounds as a thin film directly on the ATR unit and the data are presented as most characteristic absorption frequencies in $cm^{-1}$. High resolution mass spectra (HRMS) were recorded in Agilent UHPLC TOF mass spectrometer using electrospray ionization (ESI-positive) method.

## 2. Preparation of Substrates

Alkyl halides substrates reported in Table 2, entries 1, 3-6 and Table 4, entries 1-5 are commercially available.

### A. Preparation of Alkyl Bromides

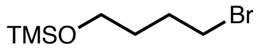


**(4-Bromobutoxy)trimethylsilane(1)**

The title compound was prepared following the procedure reported in the literature.(2)

A mixture of (4-bromobutoxy)trimethylsilane (1.00 g, 6.54 mol) and imidazole (668.5 mg, 9.81mmol) was dissolved in $CH_2Cl_2$ (30 mL) and finally TMSCl (852.6 mg, 7.85 mmol) added to the reaction mixture at room temperature. After stirring overnight, 10 mL of saturated aqueous solution of $NHCO_3$ was added and the organic layer was dried (using $NaSO_4$) filtered and concentrated under reduced pressure. The product was isolated via column chromatography (silica gel; hex: $CH_2Cl_2$ = 100:1, v/v) as pale-yellow color oil (1.33 g, 90 %). $^1$H NMR (600 MHz, $CDCl_3$) $^1$H NMR (600 MHz, $CDCl_3$-*d*) δ 3.60 (t, *J* = 6.3 Hz, 2H), 3.42 (t, *J* = 6.8 Hz, 2H), 1.92 (p, *J* = 6.9 Hz, 2H), 1.65 (p, *J* = 13.3, 6.4 Hz, 2H), 0.09 ppm (s, 9H); $^{13}$C NMR (151 MHz, $CDCl_3$) δ 61.5, 33.8, 31.2, 29.3, -0.6 ppm.

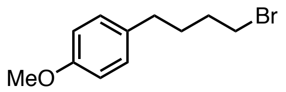


**4-(4-Methoxyphenyl)hexyl bromide(3)**

The title compound was prepared following the procedure reported in the literature.(4)

$PPh_3Br_2$ (2.81 g, 6.66 mmol) was slowly added to the solution of 4-(4-methoxyphenyl)-1-butanol (1.00 g, 4.05 mmol), and finally imidazole (453 mg, 6.66 mmol) was added after cooling the reaction mixture to 0 °C. The resulting mixture was allowed to warm to room temperature. After stirring for 19h, 10 mL of saturated aqueous solution of $Na_2S_2O_3$ and 5 mL of NaOH (6M aqueous solution) were added and the compound was extracted using $CH_2Cl_2$ (40 mL). The organic layer was dried (using $MgSO_4$) filtered and concentrated under reduced pressure. The product was isolated via column chromatography (silica gel; hex: $CH_2Cl_2$ = 6:1, v/v) as colorless oil (0.708 g, 72%). $^1$H NMR (600 MHz, $CDCl_3$) δ 7.15 – 7.10 (m, 2H), 6.89 – 6.85 (m, 2H), 3.81 (s, 3H), 3.44 (t, *J* = 6.8 Hz, 2H), 2.61 (t, *J* = 7.6 Hz, 2H), 1.91 (p, *J* = 6.9 Hz, 2H), 1.77 ppm (p, *J* = 7.6 Hz, 2H); $^{13}$C NMR (151 MHz, $CDCl_3$) δ 157.8, 133.9, 129.3, 113.8, 55.3, 34.1, 33.8, 32.3, 30.3 ppm.

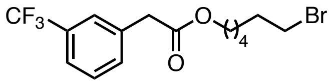

**6-Bromohexyl 2-(3- trifluoromethyl)phenyl) acetate(4)**

The title compound was prepared following the procedure reported in the literature.(4)

A mixture of 1,6-dibromohexane (4.40 g, 18.0 mmol), 2-(3-trifluoromethyl)phenyl acetic acid (1.22 g, 6.00 mmol), and $K_2CO_3$ (2.50 g, 18.0 mmol) were dissolved in acetone and refluxed for 24 h. Then the reaction mixture was cooled to room temperature, filtered through a pad Celite™ contained in a sintered glass funnel. The resulting residue was washed with acetone (~ 40 mL) and the filtrate was concentrated under reduced pressure. The resulting residue was purified using flash column chromatograph (silica gel, Hex:$CH_2Cl_2$ = 3:1, v/v) yield colorless oil (1.47 g, 67%). $^1H$ NMR (600 MHz, $CDCl_3$) δ 7.56 – 7.41 (m, 4H), 4.10 (t, *J* = 7 Hz, 2H), 3.67 (s, 2H), 3.37 (t, *J* = 7 Hz, 2H), 1.82 (p, *J* = 7.0 Hz, 2H), 1.63 (p, *J* = 8 Hz, 2H), 1.43 (p, *J* = 8 Hz, 2H), 1.32 ppm (p, *J* = 8 Hz, 2H); $^{13}C$ NMR (151 MHz, $CDCl_3$)δ 170.9, 135.1, 132.8, δ 130.9 (q, *J* = 32 Hz), 129.1, 126.2 (q, *J* = 4 Hz), 124.1 (q, *J* = 272 Hz), 124.0 (q, *J* = 4 Hz), 65.1, 41.1, 33.8, 32.6, 28.4, 27.7, 25.1 ppm.

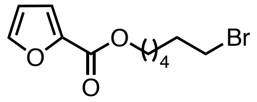

**6-Bromohexyl furan-2-carboxylate**

The title compound was prepared following the method as described for the synthesis of 6-bromohexyl 2-(3-trifluoromethyl)phenyl) acetate using furan-2-carboxylic acid (1.0 g, 8.92 mmol). The product was isolated via column chromatography (silica gel; Hex:$CH_2Cl_2$ = 3:1, v/v), as light-yellow oil (0.294 g, 11%).$^1H$ NMR (600 MHz, $CDCl_3$) δ 7.56 (dd, *J* = 1.7, 0.8 Hz, 1H), 7.16 (dd, *J* = 3.5, 0.9 Hz, 1H), 6.49 (dd, *J* = 3.5, 1.7 Hz, 1H), 4.29 (t, *J* = 6.7 Hz, 2H), 3.39 (t, *J* = 6.8 Hz, 2H), 1.86 (p, *J* = 6.8 Hz, 2H), 1.75 (p, *J* = 6.7 Hz, 2H), 1.53 – 1.36 ppm (m, 4H); $^{13}C$ NMR (151 MHz, $CDCl_3$) δ 158.8, 146.3, 144.8, 117.8, 111.8, 64.8, 33.8, 32.6, 28.5, 27.8, 25.2 ppm; FT-IR(neat) 31333, 2937, 2860, 1718, 1580, 1473, 1293, 1230, 1179, 1118, 1013, 762 $cm^{-1}$; MS (ESI) m/z $(M+Na)^+$ calculated for $C_{11}H_{15}BrO_3Na$: 297.0097, found: 297.0098.

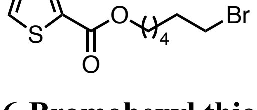

**6-Bromohexyl thiophene-2-carboxylate**

The title compound was prepared following the method as described for the synthesis of 6-bromohexyl 2-(3-trifluoromethyl)phenyl) acetate using thiophene-2-carboxylic acid (2.0 g, 15.6 mmol). The product was isolated via column chromatography (silica gel; Hex:EtOAc = 97:3, v/v) as light-yellow oil (0.693 g, 15%). $^{1}$H NMR (600 MHz, $CDCl_3$) δ 7.77 (dd, *J* = 3.7, 1.4 Hz, 1H), 7.52 (dd, *J* = 5.0, 1.4 Hz, 1H), 7.09 – 7.03 (m, 1H), 4.26 (t, *J* = 6.6 Hz, 2H), 3.38 (t, *J* = 6.8 Hz, 2H), 1.85 (p, *J* = 6.8 Hz, 2H), 1.73 (dt, *J* = 13.6, 6.6 Hz, 4H), 1.51 – 1.37 (m, 4H); $^{13}$C NMR (151 MHz, $CDCl_3$) δ 162.18, 133.90, 133.26, 132.27, 127.72, 64.94, 33.75, 32.58, 28.50, 27.76, 25.17 ppm; FT-IR(neat) 3103, 2936, 2859, 1705, 1525, 1419, 1278, 1258, 1225, 1093, 1075, 750, 721 $cm^{-1}$; MS (ESI) m/z $(M+Na)^+$ calculated for $C_{11}H_{15}BrO_2SNa$: 312.9868, found: 312.9876.

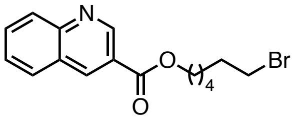

**6-Bromohexyl quinoline-3-carboxylate(4)**

The title compound was prepared following the method as described for the synthesis of 6-bromohexyl 2-(3-trifluoromethyl)phenyl) acetate using quinoline-3-carboxylic acid (1.04 g, 6.00 mmol). The product was isolated was isolated via column chromatography (silica gel; Hex:EtOAc = 5:1, v/v) as light-blue solid ( 1.00 g, 50%). $^{1}$H NMR (600 MHz, $CDCl_3$) δ 9.44 (s, 1H), 8.83 (s, 1H), 8.16 (d, *J* = 8.5 Hz, 1H), 7.94 (d, *J* = 8.2 Hz, 1H), 7.83 (t, *J* = 7.7 Hz, 1H), 7.62 (t, *J* = 7.5 Hz, 1H), 4.42 (t, *J* = 6.7 Hz, 2H), 3.43 (t, *J* = 6.7 Hz, 2H), 1.93 – 1.82 (m, 4H), 1.59 – 1.47 ppm (m, 4H); $^{13}$C NMR (151 MHz, $CDCl_3$) δ 165.5, 150.2, 149.9, 138.8, 132.0, 129.6, 129.22, 127.6, 127.0, 123.3, 65.5, 33.9, 33.0, 28.7, 27.9, 25.4 ppm.

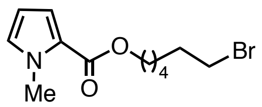

**6-Bromohexyl N-methylpyrrole-2-carboxylate(5)**

The title compound was prepared following the method as described for the synthesis of 6-bromohexyl 2-(3-trifluoromethyl)phenyl) acetate using N-metylpyrrole-2-carboxylic acid (1.00 g, 7.90 mmol). The product was isolated via column chromatography (silica gel; Hex:$CH_2Cl_2$ = 2:1, v/v) as colorless oil (1.91 g, 84%). $^{1}$H NMR (600 MHz, $CDCl_3$), δ 6.93 (dd, *J* = 3.6, 1.8 Hz, 1H), 6.79 – 6.76 (m, 1H), 6.10 (d, *J* = 2.5 Hz, 1H), 4.21 (t, *J* = 6.6 Hz, 2H), 3.91 (s, 3H), 3.40 (t, *J* = 6.8 Hz, 2H), 1.87 (p, *J* = 6.9 Hz, 2H), 1.73 (p, *J* = 7.6, 7.0 Hz, 2H), 1.53 – 1.41 ppm (m, 4H); $^{13}$C NMR (151 MHz, $CDCl_3$) δ 161.5, 129.6, 122.7, 117.8, 107.9, 63.8, 37.0, 33.9, 32.8, 28.8, 28.0, 25.4 ppm.

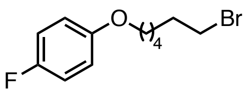

**1-[(6-Bromohexyl)oxy]-4-fluorobenzene(6)**

The title compound was prepared following the method as described for the synthesis of 6-bromohexyl 2-(3-trifluoromethyl)phenyl) acetate using 4-fluorophenol (1.00 g, 8.92 mmol). The product was isolated via column chromatography (silica gel; Hex:$CH_2Cl_2$ = 6:1, v/v) as colorless oil (1.45 g, 59%). $^1$H NMR (600 MHz, $CDCl_3$) δ 7.03 – 6.91 (m, 2H), 6.89 – 6.75 (m, 2H), 3.91 (t, $J$ = 6.4 Hz, 2H), 3.42 (t, $J$ = 6.8 Hz, 2H), 1.96 – 1.83 (m, 2H), 1.82 – 1.75 (m, 2H), 1.56 – 1.41 (m, 4H); $^{13}$C NMR (151 MHz, $CDCl_3$) δ 157.3 (d, $J$ = 237.8 Hz), 155.3 (d, $J$ = 2.1 Hz), 115.8 (d, $J$ = 23.1 Hz), 115.5 (d, $J$ = 7.8 Hz), 68.5, 33.9, 32.8, 29.2, 28.0, 25.4 ppm.

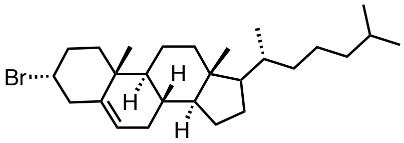

**3$\alpha$-Bromocholest-5-ene(7)**

3$\alpha$-Bromocholest-5-ene was prepared following the procedure reported in the literature.(8)

A mixture of cholesterol (193 mg, 0.50 mmol) and $PPh_3$ (197 mg, 0.75 mmol) was dissolved in dichloromethane (10 mL) at ambient temperature. Then $CBr_3$ (182 mg, mmol) was added to the reaction mixture in portion and stirred overnight at room temperature. The solvent was removed by vacuo and the residue was purified via column chromatography (silica gel; hexane) as white solid (0.185 g, 82%). $^1$H NMR (600 MHz, $CDCl_3$) δ 5.52 – 5.26 (m, 1H), 3.96 – 3.89 (m, 1H), 2.83 – 2.53 (m, 2H), 2.25 – 2.11 (m, 1H), 2.09 – 1.91 (m, 3H), 1.89 – 1.73 (m, 2H), 1.63 – 1.21 (m, 11H), 1.18 – 0.89 (m, 15H), 0.86 (dd, $J$ = 6.6, 2.8 Hz, 6H), 0.67 ppm (s, 3H); $^{13}$C NMR (151 MHz, $CDCl_3$) δ 141.7, 122.5, 56.8, 56.3, 52.8, 50.3, 44.4, 42.4, 40.5, 39.8, 39.7, 36.5, 36.3, 35.9, 34.5, 31.95, 31.85, 28.4, 28.2, 24.4, 24.0, 23.0, 22.7, 21.0, 19.4, 18.9, 12.0 ppm.

## B. Preparation of Alkyl Tosylates

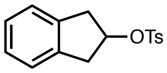

**2, 3-Dihydro-1*H*-inden-2-yl 4-methylbenzenesulfonate(9)**

The title compound was prepared following the procedure reported in the literature.(4)

A mixture of 2-indanol (671 mg, 5.0 mmol) and pyridine (0.80 mL, 10.0 mmol) was dissolved in

$CH_2Cl_2$ (15 mL), and cooled to 0 °C. Finally, tosyl chloride (1.05 g, 5.50 mmol) was slowly added to the cold reaction mixture and was allowed to warm to room temperature. After 17 h, saturated aqueous solution of $NaHCO_3$ (10 mL) was added and the aqueous layer was extracted with $CH_2Cl_2$ (1 x 20 mL). The combined organic phase was dried over $MgSO_4$, filtered, and concentrated under reduced pressure. The resulting crude residue was subjected to flash chromatography (silica gel, hexane/ EtOAc = 10:1, v/v) The title compound was obtained as a colorless solid in (1.12 g, 78%). $^1H$ NMR (600 MHz, $CDCl_3$) δ 7.87 – 7.67 (m, 2H), 7.40 – 7.30 (m, 2H), 7.19 -7.15 (m, 4H), 5.29 (tt, *J* = 6.3, 3.8 Hz, 1H), 3.30 – 3.02 (m, 4H), 2.46 ppm (s, 3H); $^{13}C$ NMR (151 MHz, $CDCl_3$) δ 144.9, 139.3, 134.2, 130.0, 127.9, 127.2, 124.7, 82.6, 39.90, 21.8, 0.2 ppm.

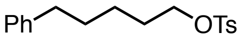


**Benzenepentanol, 4-methylbenzenesulfonate(10)**

The title compound was prepared following the method as described for the synthesis of 2,3-dihydro-1*H*-inden-2-yl 4-methylbenzenesulfonate using 5-phenyl-1-pentanol (411 mg, 2.50 mmol). The product was isolated via column chromatography (silica gel; hex: EtOAc = 10:1, v/v) as colorless oil (0.490 g, 61%). $^1H$ NMR (600 MHz, $CDCl_3$) δ 7.80 – 7.73 (m, 2H), 7.32 (dt, *J* = 7.9, 0.8 Hz, 2H), 7.27 – 7.23 (m, 2H), 7.19 – 7.14 (m, 1H), 7.13 – 7.09 (m, 2H), 4.00 (t, *J* = 6.5 Hz, 2H), 2.54 (t, *J* = 7.7 Hz, 2H), 2.43 (s, 3H), 1.65 (dt, *J* = 14.3, 6.7 Hz, 2H), 1.55 (p, *J* = 7.7 Hz, 2H), 1.33 ppm (p, *J* = 7.7 Hz, 2H); $^{13}C$ NMR (151 MHz, $CDCl_3$) δ 144.8, 142.2, 133.1, 129.9, 128.4, 128.3, 127.9, 125.8, 70.6, 35.7, 30.8, 28.7, 25.0, 21.7 ppm.

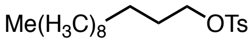


**1-Dodecyl-methylbenzenesulfonate(4)**

The title compound was prepared following the method as described for the synthesis of 2,3-dihydro-1*H*-inden-2-yl 4-methylbenzenesulfonate using dodecan-1-ol (932 mg, 5.00 mmol). The product was isolated via column chromatography using silica gel (hex: $CH_2Cl_2$ = 2:1, v/v) as colorless oil (1.08 g, 69%). $^1H$ NMR (600 MHz, $CDCl_3$) δ 7.81 – 7.77 (m, 2H), 7.35 – 7.33 (m, 2H), 4.01 (t, *J* = 6.5 Hz, 2H), 2.45 (s, 3H), 1.62 (p, *J* = 6.8 Hz, 2H), 1.31 – 1.17 (m, 18H), 0.88 ppm (t, *J* = 7.0 Hz, 3H); $^{13}C$ NMR (151 MHz, $CDCl_3$) δ 144.7, 133.3, 129.9, 128.0, 70.9, 32.0Table, 29.75, 29.63, 29.53, 29.48, 29.1, 28.9, 25.5, 22.8, 21.8, 14.3 ppm.

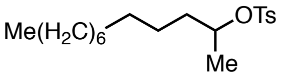


**2-Dodecyl-methylbenzenesulfonate(4)**

The title compound was prepared following the method as described for the synthesis of 2,3-dihydro-1*H*-inden-2-yl 4-methylbenzenesulfonate using dodecan-2-ol (932 mg, 5.00 mmol). The product was isolated via column chromatography using silica gel (hex: $CH_2Cl_2$ = 2:1, v/v) as colorless oil (0.920 g, 59%). $^1$H NMR (600 MHz, $CDCl_3$) δ 7.82 – 7.76 (m, 2H), 7.34 – 7.31 (m, 2H), 4.63 – 4.55 (m, 1H), 2.44 (s, 3H), 1.65 – 1.42 (m, 2H), 1.33 – 1.09 (m, 18H), 0.88 ppm (t, *J* = 7.0 Hz, 3H); $^{13}$C NMR (151 MHz, $CDCl_3$) δ 144.5, 134.7, 129.8, 127.9, 80.9, 36.6, 32.0, 29.72, 29.63, 29.55, 29.47, 29.28, 25.0, 22.8, 21.8, 21.0, 14.3 ppm.

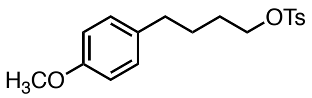


**4-(4-Methoxyphenyl)butyl 4-methylbenzesulfonate(5)**

The title compound was prepared following the method as described for the synthesis of 2,3-dihydro-1*H*-inden-2-yl 4-methylbenzenesulfonate using 4-(4-methoxyphenyl)-1-butanol (451mg, 2.50 mmol). The product was isolated via column chromatography using silica gel (hex: $CH_2Cl_2$ = 2:1, v/v) as colorless oil (0.352 g, 42%). $^1$H NMR (600 MHz, $CDCl_3$) δ 7.83 – 7.72 (m, 2H), 7.37 – 7.30 (m, 2H), 7.05 – 6.97 (m, 2H), 6.83 – 6.77 (m, 2H), 4.03 (t, *J* = 6.2 Hz, 3H), 3.78 (s, 3H), 2.50 (t, *J* = 7.4 Hz, 2H), 2.44 (s, 3H), 1.72 – 1.54 ppm (m, 4H); $^{13}$C NMR (151 MHz, $CDCl_3$) δ 157.9, 144.8, 133.7, 133.2, 129.9, 129.3, 127.9, 113.8, 70.6, 55.3, 34.2, 28.3, 27.4, 21.7 ppm.

**C. Preparation of Alkyl Mesylates**

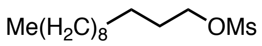


**1-(Methanesulfonyloxy)dodecane(11)**

The title compound was prepared was prepared following the procedure reported in the literature.(4)

A mixture of decan-1-ol (3.00 mL, 13.4 mmol), $Et_3N$ (5.60 mL, 40.2 mmol), and DMAP (40.0 mg, 0.330 mmol) was dissolved in $CH_2Cl_2$ (30 mL) and cooled to 0 °C. To the cold reaction mixture, mesyl chloride (2.00 mL, 26.8 mmol) was slowly added was allowed to slowly warm to room temperature. After 16 h, $NaHCO_3$ (saturated aqueous solution; 10 mL) was added, the aqueous layer was extracted with $CH_2Cl_2$ (2 x 20 mL). The combined organic phase was dried ($MgSO_4$), filtered, and concentrated under reduced pressure. The resulting crude residue was subjected to flash chromatography (hex: $CH_2Cl_2$ = 4:1). The product was purified via column chromatography using silica gel (hex: $CH_2Cl_2$ = 4:1, v/v) as colorless oil (2.60 g, 82%). $^1$H NMR (600 MHz, $CDCl_3$)

δ 4.22 (t, $J$ = 6.6 Hz, 2H), 3.00 (s, 2H), 1.78 – 1.69 (m, 2H), 1.43 – 1.35 (m, 2H), 1.34 – 1.19 (m, 16H), 0.88 ppm (t, $J$ = 7.0 Hz, 3H); $^{13}$C NMR (151 MHz, $CDCl_3$) δ 70.4, 37.5, 32.0, 29.75, 29.66, 29.56, 29.5, 29.3, 29.2, 25.6, 22.8, 14.3 ppm.

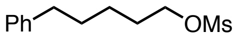


**Benzenepentanol, 1-methyanesulfonate(12)**

The title compound prepared following the method as described for the synthesis of 1-(methanesulfonyloxy)dodecane using 5-phenyl-1-pentanol (401 mg, 2.44 mmol). The product was purified via column chromatography using silica gel (hex: EtOAc = 10:1, v/v) as colorless solid (0.453 g, 77%). $^{1}$H NMR (600 MHz, $CDCl_3$) δ 7.34 – 7.26 (m, 2H), 7.23 – 7.15 (m, 2H), 4.22 (t, $J$ = 6.6 Hz, 2H), 2.98 (s, 3H), 2.64 (t, $J$ = 7.9 Hz, 2H), 1.79 (p, $J$ = 6.9 Hz, 2H), 1.68 (p, $J$ = 7.9 Hz, 2H), 1.46 ppm (p, $J$ = 7.8 Hz, 2H); $^{13}$C NMR (151 MHz, $CDCl_3$) δ 142.2, 128.41, 128.37, 125.8, 70.1, 37.3, 35.7, 30.9, 29.0, 25.0 ppm.

## 3. Optimization of $VB_{12}$-Photocatalyzed Olefination of Alkyl halide

In order to optimize the reaction condition of $VB_{12}$-photocatalytic dehydrohalogenation reaction, 1-chlorooctane was used as the model substrate. All the control reactions (Table S1, entries 1-14) were set-up at 0.1 mmol scale and in 0.1 molar concentration (except Table S1, entry 14) at room temperature. Terminal alkene product was obtained in all the control reactions, the isomerized (semiterminal) product was notably absent in all the control reactions where the yields are specified. The catalytic reaction did not proceed forward in the absence of light, reductant and base (Table S1, entries 2-5). No reaction was observed when reductants such as phenylsilane, $B_2Pin_2$, zinc (Zn) and manganese (Mn) were used. Further, no reaction occurred when a weak inorganic base $NaHCO_3$ was substituted by equal amount of triethylamine (Table S1, entry 6). Interestingly, when the amount of $NaBH_4$ was lowered from 4.0 equivalents to 1.5 equivalent, the efficiency of the reaction was significantly reduced, only producing 56% of the desired product (Table S1, entry 7). Various cobalt pre-catalysts were investigated, it was found that AdoCbl was unsuccessful to catalyze the reaction (Table S1, entries 8), whereas cobaloxime pyridine chloride (COPC) produced some desired product **3a** in 34% yield (Table S1, entrie 9). Among the various solvents screened, acetonitrile was found to the best that gave desired terminal alkene selectively. Low selectivity was observed upon using dimethylforamide (DMF), with the yield of desired terminal alkene **3a** decreasing to 35% and remainder of the mass balance corresponding to side product **3b** (Table S1 entry 10). Similarly, the catalytic action of our method was greatly reduced by the polar solvents dimethyl sulfoxide (DMSO) and acetone, favoring the formation of reduced side product **3b,** with olefinic product **3a** being formed in 17% and 21% respectively (Table S1, entries 11-12). Furthermore, rate of the reaction was slower when the catalyst loading of **C2** was reduced from 5

mol% to 2.5 mol% (Table S1, entry 13) and with the decrease in concentration of the reaction (Table S1, entry 14). Finally, we investigated the effect of some common mild bases under our optimized reaction condition, and upon using $Na_2CO_3$, only 36% of desired product was formed with only 50% conversion (Table S1, entry 15). Whereas the mild base $K_2CO_3$ was able to drive the reaction to completion producing low yield of the desire terminal alkene (Table S1, entry 16).

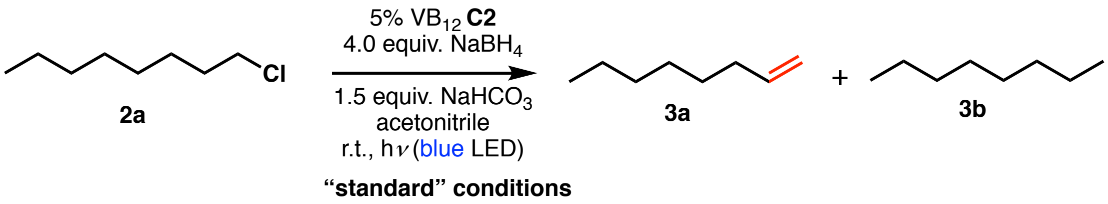


| entry | variation from the optimized conditions | time | % yield (3a)[a] | % conversion |
|---|---|---|---|---|
| **1** | **none** | **16 h** | **75** | 100[b] |
| 2 | no light | 2 d | 0 | 0[c] |
| 3 | no **C2** or no $NaHCO_3$ | 2 d | 0 | 0[c] |
| 4 | phenylsilane or $B_2Pin_2$ instead of $NaBH_4$ | 2 d | 0 | 0[c] |
| 5 | Zn or Mn instead of $NaBH_4$ | 22 h | 0 | 0[c] |
| 6 | triethlyamine instead of $NaHCO_3$ | 2 d | 0 | 0[c] |
| 7 | 1.5 equiv. instead of 4.0 equiv. $NaBH_4$ | 16 h | 56 | 100[b] |
| 8 | 5% CoPC instead of 5% C2 | 21h | 0 | 100[d] |
| 9 | 5% AdoCbl **C1** instead of 5% C2 | 15 h | 34 | 100[b] |
| 10 | DMF instead of acetonitrile | 16 h | 35 | 100[b] |
| 11 | DMSO instead of acetonitrile | 24 h | 17 | 100[b] |
| 12 | acetone instead of acetonitrile | 24 h | 21 | 100[b] |
| 13 | 2.5 mol% **C2** loading | 17 h | N/A | 74 |
| 14 | reaction using 0.05M concentration | 16 h | N/A | 61 |
| 15 | 1.0 equiv. $Na_2CO_3$ instead of 1.5 equiv. $NaHCO_3$ | 18 h | 36 | 50[b] |
| 16 | 1.5 equiv. $K_2CO_3$ instead of 1.5 equiv. $NaHCO_3$ | 20 h | 35 | 100[b] |

[a] Light irradiation [ LED, $\lambda_{max}$ = 427 nm]. Determination of yields via NMR using 1,3,5-trimethoxybenzene as an internal standard. All reactions were carried out using **2a** applying standard conditions (0.1 mmol) in 0.1M concentration under a $N_2$ atmosphere at room temprature.
[b] Remaining mass balanced by side product **3b**
[c] Only starting material
[d] ~ 40% internal alkene and **3b** side product

**Table S1**: Control experiments conducted to optimize dehydrohalogenation reaction using 1-chlorooctane.

## 4. General Method of $B_{12}$ Catalyzed Dehydrogenation

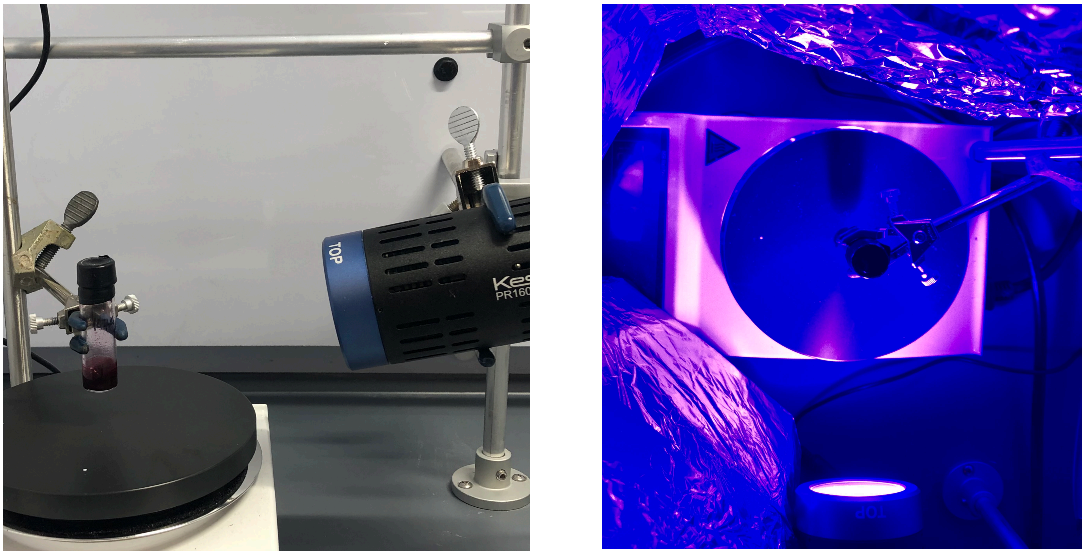


**Figure S1**: Photographs of reaction set-up: a) The side view of reaction set-up before operation. The Kessil LED lamp ($\lambda_{max}$ = 427) is placed 15 cm away from the reaction vial (left); b) The overhead view of reaction set-up during operation (right).

$VB_{12}$ **C2** (6.80 mg, 0.005 mmol, 5.0 mol%), $NaBH_4$ (15.1 mg, 0.399 mmol, 4.0 equiv.), $NaHCO_3$ (12.6 mg, 0.149 mmol, 1.2 equiv.), internal standard 1,3,5- trimethoxybenzene (2.0 - 5.0 mg) and a stir bar were added to an oven-dried 8-mL glass vial. A rubber cap was fitted to the vial, which was then evacuated and backfilled with nitrogen (3 cycles). The vial was detached from the nitrogen line, and 1mL of acetonitrile was added via syringe. Then the nitrogen gas was bubbled through the reaction mixture for about 10 min and alkyl halide substrate (0.10 mmol) was added and nitrogen was bubbled again for 5 min. The reaction mixture was placed under blue light after sealing the punctured holes of the vial cap with vacuum grease and electric tape. The reaction mixture was stirred for 16 h, and then about 0.1ml of the reaction mixture was filtered through silica (using short pipet column) to remove solid residue. The silica was washed with $CDCl_3$ (0.1 mL). The filtrate was then washed with DI water and the lower $CDCl_3$ layer was taken for $^1H$ NMR spectroscopy after drying with $MgSO_4$.

## 5. General Method of Remote Elimination

$VB_{12}$ **C2** (6.80 mg, 0.005 mmol, 5.0 mol%), cobalt salen complex **C4** (3.00 mg, 0.005 mmol, 5.0 mol%) $NaBH_4$ (15.1 mg, 0.399 mmol, 4.0 equiv.), $NaHCO_3$ (12.6 mg, 0.149 mmol, 1.2 equiv.), internal standard 1,3,5- trimethoxybenzene (2.0 - 5.0 mg) and a stir bar were added to an oven-dried 8-mL glass vial. A rubber cap was fitted to the vial, which was then evacuated and backfilled with nitrogen (3 cycles). The vial was detached from the nitrogen line, and 1mL of acetonitrile

was added via syringe. Then the nitrogen gas was bubbled through the reaction mixture for about 10 min and alkyl sulfonate substrate (0.10 mmol) was added and nitrogen was bubbled again for 5 min. The reaction mixture was placed under blue light after sealing the punctured holes of the vial cap with vacuum grease and electric tape. The reaction mixture was stirred for 16 h, and then about 0.1ml of the reaction mixture was filtered through silica (using short pipet column) to remove solid residue. The silica was washed with $CDCl_3$ (0.1 mL). The filtrate was then washed with DI water and the lower $CDCl_3$ layer was taken for $^1$H NMR spectroscopy after drying with $MgSO_4$.

**Note:** In the dehydrohalogenation and remote elimination reactions, some corresponding alkane as side product was absorbed which made the isolation of the desired product difficult because of their similar $R_f$. Therefore, the yield of the target compounds have been reported based on the mmol of internal standard, i.e. 1,3,5, trimethoxybenzene (all the target compounds are known and previously characterized).

The products of substrates Table 2, entries 1, 3-6, Table 4, entries 1, 4 and 5 are identical to authentic material (Aldrich, Oakwood, TCI) by $^1$HNMR spectroscopy.

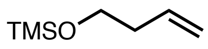


3-Butenyloxytrimethylsilane (Table 2, entry 2)

The title compound was synthesized using procedure described in general method 4. The NMR yield of the product was calculated using 1, 3, 5-trimethoxybenzene as an internal standard and was found to be 60 % yield. The compound was identical to the compound reported in the literature by proton NMR.(13)

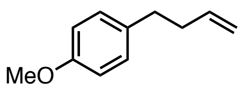


**4-(4-methoxy phenyl)-1-butene** (Table 2, entry 7)

The title compound was synthesized using procedure described in general method 4. The NMR yield of the product was calculated using 1, 3, 5-trimethoxybenzene as an internal standard and was found to be 71% yield. The compound was identical to the compound reported in the literature by proton NMR.(14)

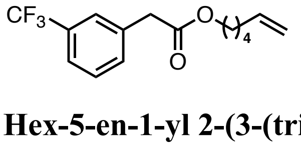


**Hex-5-en-1-yl 2-(3-(trifluoromethyl)phenyl)acetate** (Table 2, entry 8)

The title compound was synthesized using procedure described in general method 4. The NMR yield of the product was calculated using 1, 3, 5-trimethoxybenzene as an internal standard and was found to be 57% yield. The compound was identical to the compound reported in the literature by proton NMR.(4)

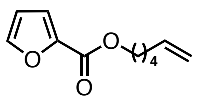

**Hex-5-en-1-yl furan-2-caboxylate** (Table 2, entry 9)

The title compound was synthesized procedure described in general method 4. The NMR yield of the product was calculated using 1,3,5-trimethoxybenzene as an internal standard and was found to be 61% yield. The compound was identical to the compound reported in the literature by proton NMR. (15)

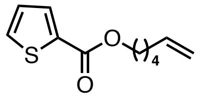

**Hex-5-en-1-yl thiophene-2-caboxylate** (Table 2, entry 10)

The title compound was synthesized using procedure described in general method 4. The NMR yield of the product was calculated using 1,3,5-trimethoxybenzene as an internal standard and was found to be 66% yield. The compound was identical to the compound reported in the literature by proton NMR.(15)

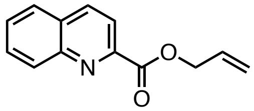

**Hexyl-5-en-1-yl quinoline-3-carboxylate** (Table 2, entry 11)

The title compound was synthesized using procedure described in general method 4. The NMR yield of the product was calculated using 1,3,5-trimethoxybenzene as an internal standard and was found to be 53% yield. The compound was identical to the compound reported in the literature by proton NMR.(4)

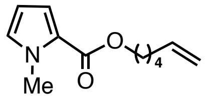

**Hexyl-5-en-1-yl *N*-methylpyrrole-2-carboxylate** (Table 2, entry 12)

The title compound was synthesized using procedure described in general method 4. The NMR yield of the product was calculated using 1,3,5-trimethoxybenzene as an internal standard and was found to be 69% yield. The compound was identical to the compound reported in the literature by proton NMR.(16)

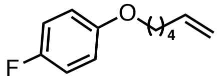

**1-(Hex-5-en-1-yloxy)-4-fluorobenzene** (Table 2, entry 13)

The title compound was synthesized using procedure described in general method 4. The NMR yield of the product was calculated using 1,3,5-trimethoxybenzene as an internal standard and was found to be 55% yield. The compound was identical to the compound reported in the literature by proton NMR.(17)

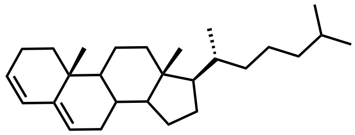

**3,5-Cholestadiene** (Table 2, entry 14)

The title compound was synthesized using procedure described in general method 4. The NMR yield of the product was calculated using 1,3,5-trimethoxybenzene as an internal standard and was found to be 52% yield. The compound was identical to the compound reported in the literature by proton NMR. (18)

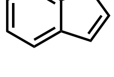

**1*H*-Indene** (Table 3, entry 1)

The title compound was synthesized using procedure described in general method 4. The NMR yield of the product was calculated using 1,3,5-trimethoxybenzene as an internal standard and was found to be 87% yield. The compound was identical to the compound reported in the literature by proton NMR.(19)

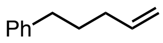


**Pent-4-en-1-ylbenzene** (Table 3, entry 2)

The title compound was synthesized using procedure described in general method 4. The NMR yield of the product was calculated using 1,3,5-trimethoxybenzene as an internal standard and was found to be 54% yield. The compound was identical to the compound reported in the literature by proton NMR.(20)

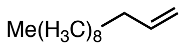


**1-Dodecene** (Table 3, entry 3)

The title compound was synthesized using procedure described in general method 4. The NMR yield of the product was calculated using 1,3,5-trimethoxybenzene as an internal standard and was found to be 56% yield. The compound was identical to the compound reported in the literature by proton NMR.(4)

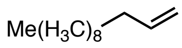


**1-Dodecene** (Table 3, entry 4)

The title compound was synthesized using procedure described in general method 4. The NMR yield of the product was calculated using 1,3,5-trimethoxybenzene as an internal standard and was found to be 56% yield. The compound was identical to the compound reported in the literature by proton NMR.(4)

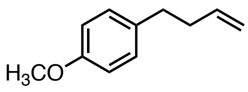


**1-(But-3-ene-1-yl)-4-methoxybenzene** (Table 3, entry 5)

The title compound was synthesized using procedure described in general method 4. The NMR yield of the product was calculated using 1,3,5-trimethoxybenzene as an internal standard and was found to be 75% yield. The compound was identical to the compound reported in the literature by proton NMR.(14)

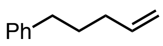

**Pent-4-en-1-ylbenzene** (Table 3, entry 6)

The title compound was synthesized using procedure described in general method 4. The NMR yield of the product was calculated using 1,3,5-trimethoxybenzene as an internal standard and was found to be 88% yield. The compound was identical to the compound reported in the literature by proton NMR.(20)

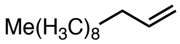


**1-Dodecene** (Table 3, entry 7)

The title compound was synthesized using procedure described in general method 4. The NMR yield of the product was calculated using 1,3,5-trimethoxybenzene as an internal standard and was found to be 53% yield. The compound was identical to the compound reported in the literature by proton NMR.(4)

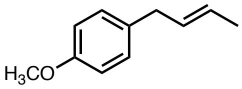


**1-(4-Methoxyphenyl)but-2-ene** (Table 4, entry 6)

The title compound was synthesized using procedure described in general method 5. The NMR yield of the product was calculated using 1,3,5-trimethoxybenzene as an internal standard and was found to be 41% yield. The compound was identical to the compound reported in the literature by proton NMR.(21)

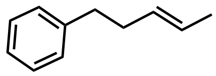

**3-Penten-1-ylbenzene** (Table 4, entry 7)

The title compound was synthesized using procedure described in general method 5. The NMR yield of the product was calculated using 1,3,5-trimethoxybenzene as an internal standard and was found to be 52% yield. The compound was identical to the compound reported in the literature by proton NMR.(22)

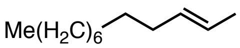


**2-Dodecene** (Table 4, entry 8)

The title compound was synthesized using procedure described in general method 5. The NMR yield of the product was calculated using 1,3,5-trimethoxybenzene as an internal standard and was found to be 66% yield. The compound was identical to the compound reported in the literature.(23)

## 6. Mechanistic Studies

### A. Inhibition Experiments

### i) TEMPO Inhibition Experiments

In a 8 ml flame dried vial, $VB_{12}$ **C2** (13.6 mg, 0.01 mmol, 5.0 mol%), $NaBH_4$ (30.3 mg, 0.80 mmol, 4.0 equiv.), $NaHCO_3$ (25.2 mg, 0.290 mmol, 1.2 equiv.), internal standard (1,3,5-trimethoxybenzene, 4.80 mg, 0.030 mmol) and a stir bar were added. A rubber cap was fitted to the vial, which was then evacuated and backfilled with nitrogen (3 cycles). The vial was detached from the nitrogen line, and 2 mL of deuterated acetonitrile was added via syringe and the nitrogen gas was bubbled through the reaction mixture for about 10 min. Then, 1.0 equiv. TEMPO (31.2 mg, 0.20 mmol) was added to the reaction vial and bubbled $N_2$ for 5 min. Finally, the 1-bromooctane substrate (0.03 ml, 0.20 mmol) was added to the reaction vial with the help of syringe. The reaction mixture was placed under blue light after sealing the holes of the vial cap with vacuum grease and electric tape. The reaction was monitored at different times shown in the table below. An aliquot of the reaction was drawn using syringe and passed through a short silica plug and diluted with ACN-$d_3$. and the reaction was monitored via NMR. The yields were calculated using 1, 3, 5-trimethoxybenzene as an internal standard. The 1-octene **A** product was identical to authentic material (Aldrich) by $^1$H NMR spectroscopy.

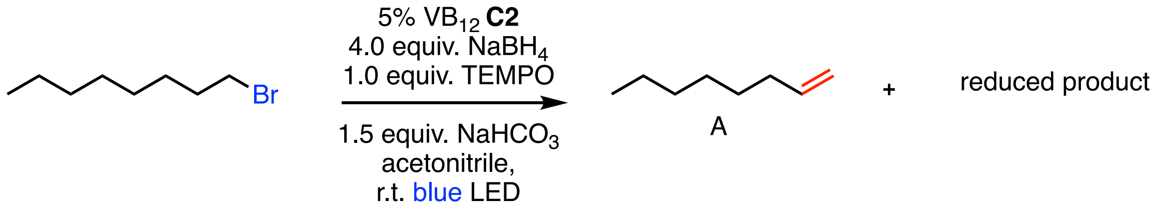


| Time | Terminal alkene A | % Conversion |
|---|---|---|
| 2 hr | 2% | 33 |
| 6 hr | 8% | 63 |
| 8 hr | 21% | 100 |

**Table S2:** Mechanistic experiment using 1-bromooctane using TEMPO under standard reaction condition.

### ii) 1,1-Diphexylethylene Inhibition Experiment

The reaction was set up following the standard method as described in the TEMPO inhibition experiment in dry acetonitrile using 1-bromooctane substrate (0.03 ml, 0.20 mmol) and 1,1-Diphenylhexylethylene (18.0 mg, 0.10 mmol). An aliquot of the reaction was drawn using syringe and passed through a short silica plug and diluted with $CDCl_3$ and NMR was taken after aqueous workup and drying with $Na_2SO_4$. The yield was calculated using 1, 3, 5-trimethoxybenzene as an internal standard. The 1-octene **A** product was identical to authentic material (Aldrich) by $^1$H NMR spectroscopy.

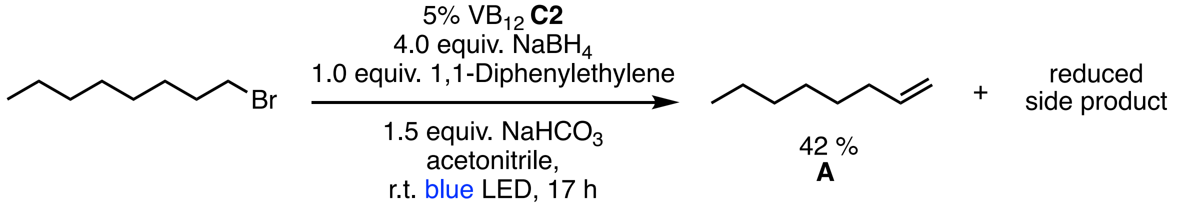


**Scheme S1:** Mechanistic experiment using 1-bromooctane and 1,1-Diphenylhexylethylene under standard reaction condition.

### iii) BHT Inhibition Experiment

The reaction was set up following the standard method as described in the TEMPO inhibition experiment using 1-bromooctane substrate (0.03 ml, 0.20 mmol) and 2,6-Di-tert-butyl-4-methylphenol (22.0 mg, 0.10 mmol). An aliquot of the reaction was drawn using syringe and passed through a short silica plug and diluted with $CDCl_3$ and NMR was taken after aqueous workup and drying with $Na_2SO_4$. The yield was calculated using 1, 3, 5-trimethoxybenzene as an internal standard. The 1-octene **A** product was identical to authentic material (Aldrich) by $^1$H NMR spectroscopy.

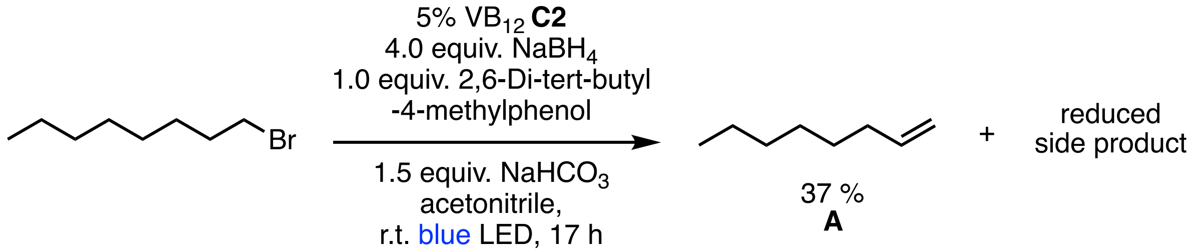


**Scheme S2:** Mechanistic experiment using 1-bromooctane and 2,6-Di-tert-butyl-4-methylphenol under standard reaction condition.

## B. Radical Clock Experiment

The reaction was set-up following the procedure described in the general method **3** using 6-bromo-1-hexene (16.3 mg, 0.100 mmol) as substrate. The reaction went to completion after 20 h to form 5-hexadiene **A** (52%) along with reduced side product **B** (32%) as shown in the Scheme S1. In this transformation, no trace of cyclic product was observed supporting our proposed reaction mechanism showing evidence of the rapid Co(II)-perpetrated HAT step in the catalytic cycle. The NMR yields were calculated using 1,3,5-trimethoxybenzene as an internal standard.

The product **A** is identical to the literature value by $^1$H NMR spectroscopy.(24)

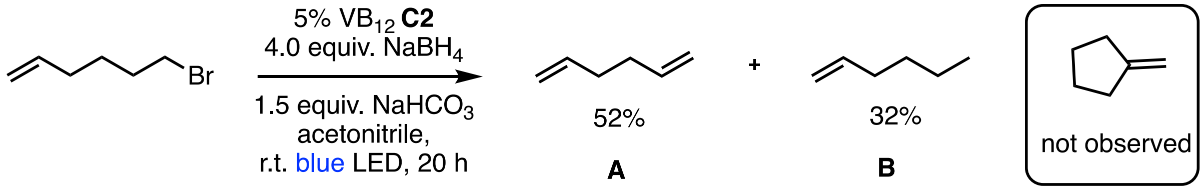


**Scheme S3:** The mechanistic study showing the formation of hexadiene product over cyclic product.

## C. Isotope Labeling Experiment

The isotope labeling experiments were conducted following the procedure described in general method **3** using 1-bromo-3-phenylpropane (19.9 mg, 0.10 mmol) as substrate and 4 equiv. of $NaBD_4$ (16.7 mg, 0.40 mmol). After 22h, from $^1$H NMR analysis; 48% of starting material was left unreacted and 3-phenylpropene product formed was calculated to be 48% (Scheme S2a). Whereas, when the same substrate was subjected to the reaction procedure described in general method **4** (remote elimination) both the subterminal alkene and terminal alkenes in 29% each along with 40% unreacted starting material (Scheme S2b). The NMR yield were obtained using 1,3,5-trimethoxybenzene as internal standard. No deuterium incorporation in the final products was observed. The *trans*-1-Phenyl-1-propene product and allylbenzene products were identical to authentic material (Aldrich) by $^1$H NMR spectroscopy.

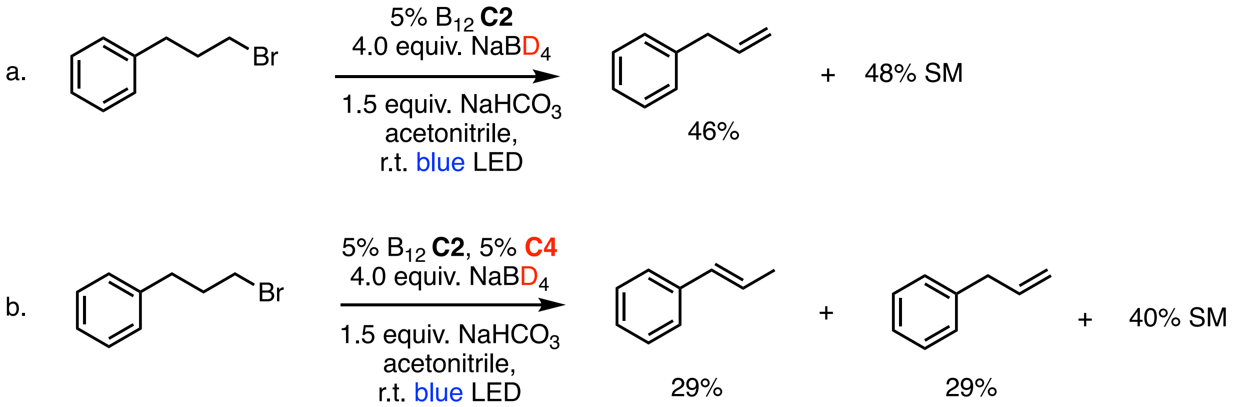


**Scheme S4:** a) Isotope labeling experiment under standard dehydrohalogenation condition; b) Isotope labeling experiment under standard remote elimination condition.

# $^{1}H$ and $^{13}C$ NMR Spectra of New Compounds

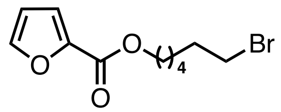


**Table 2, Entry 9**
**$CDCl_3$, NMR (600 Hz)**

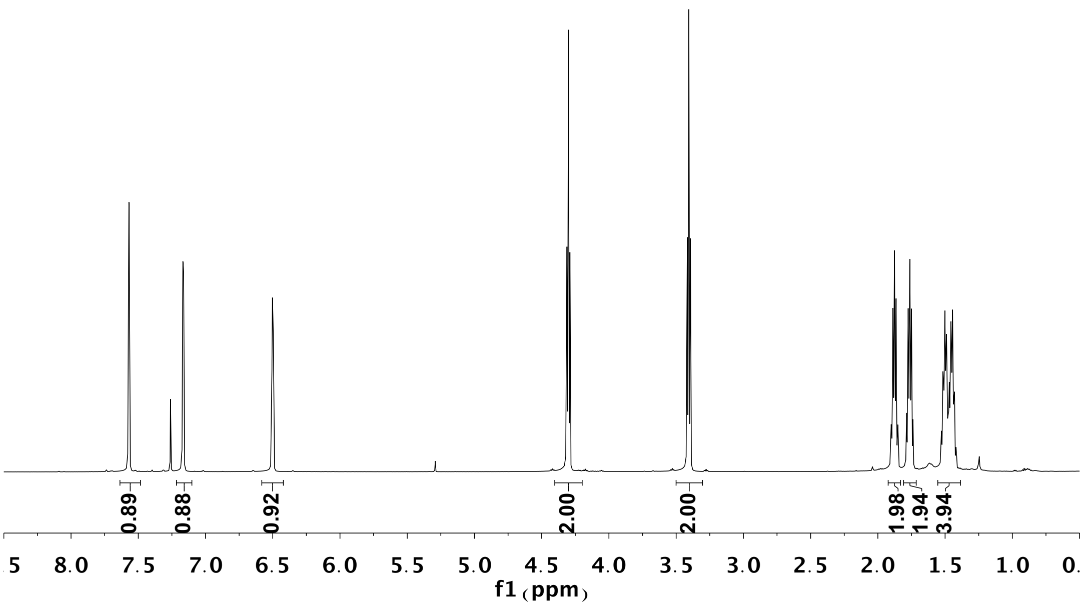


**Figure S2**: $^{1}H$ NMR spectrum for 6-bromohexyl furan-2-carboxylate in $CDCl_3$ at 298 K.

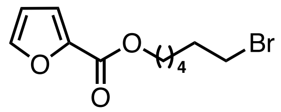

**Table 2, Entry 9**
**$CDCl_3$, NMR (600 Hz)**

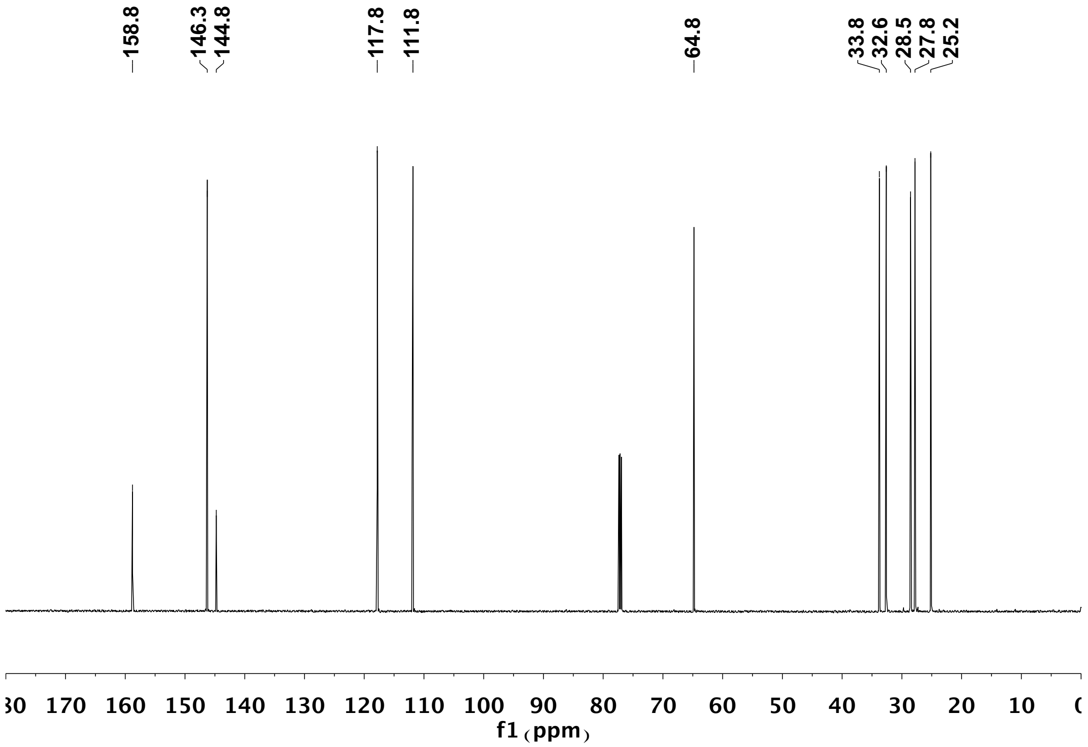


**Figure S3**: $^{13}C$ NMR spectrum for 6-bromohexyl furan-2-carboxylate in $CDCl_3$ at 298 K.

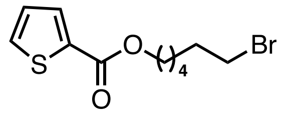


**Table 2, Entry 10**
**$CDCl_3$, NMR (600 Hz)**

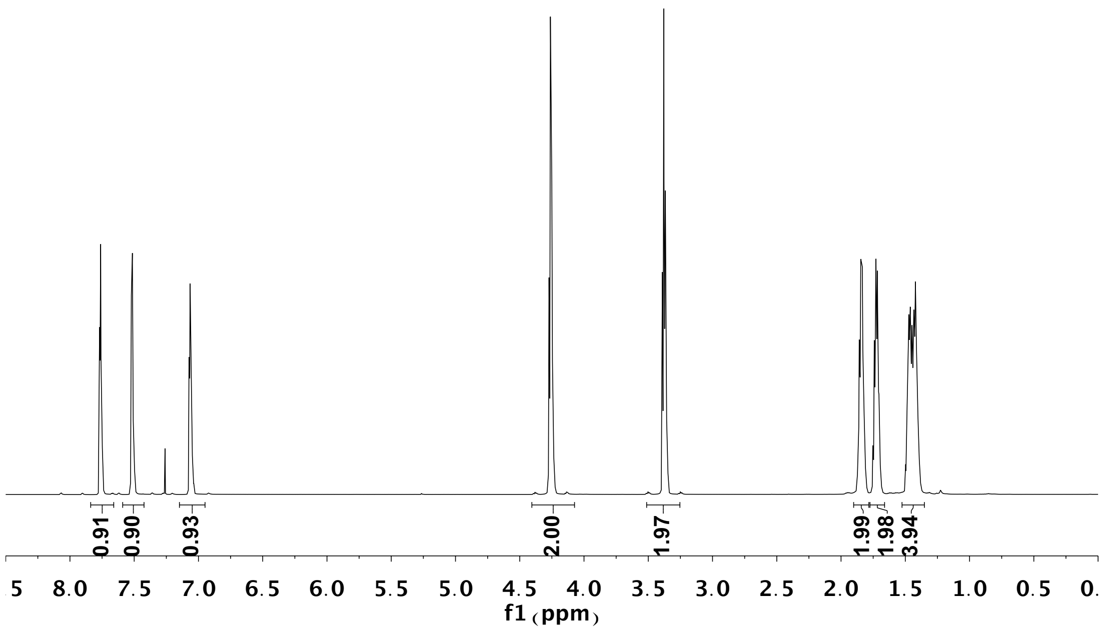


**Figure S4**: $^{1}H$ NMR spectrum for 6-bromohexyl thiophene-2-carboxylate in $CDCl_3$ at 298 K.

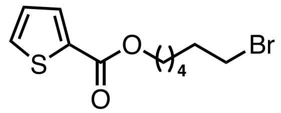


**Table 2, Entry 10**
**CDCl3, NMR (600 Hz)**

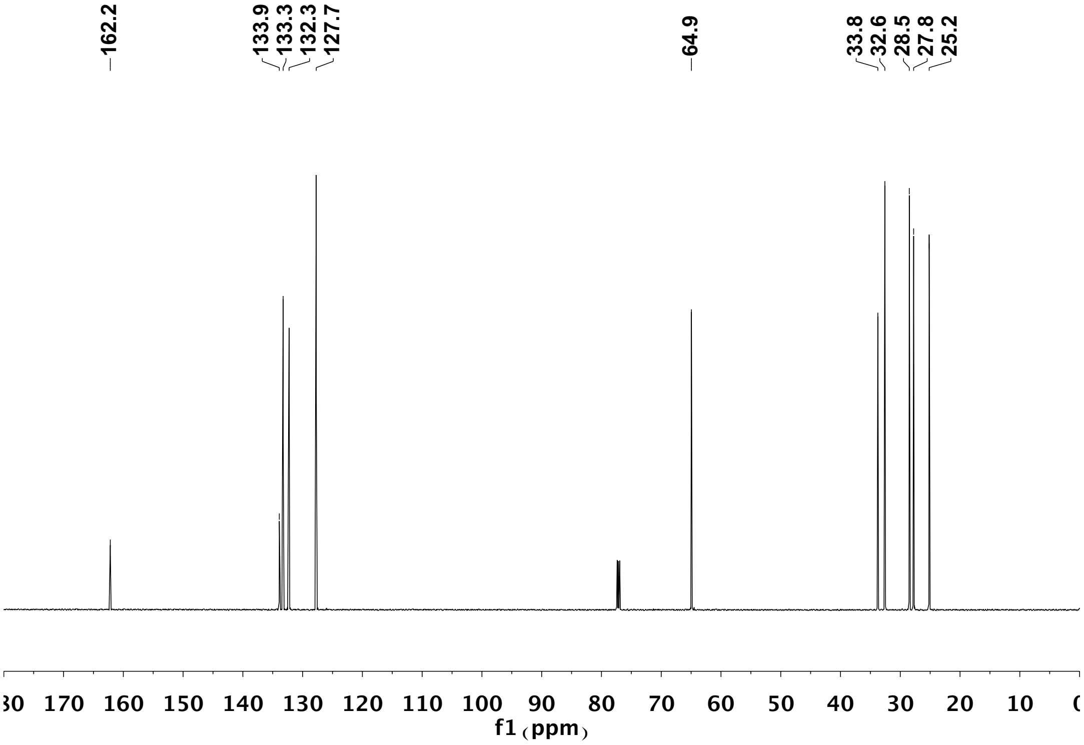


**Figure S5**: ${}^{13}C$ NMR spectrum for 6-bromohexyl thiophene-2-carboxylate in $CDCl_3$ at 298 K.

## References


1. J. Ohshita, *et al.*, Ring-Opening Iodo- and Bromosilation of Cyclic Ethers by Treatment with Iodo- and Bromotrialkylsilane Equivalents. *J. Org. Chem.* **64**, 8024–8026 (1999).

2. H. Tan, H. Liu, X. Chen, H. Chen, S. Qiu, Racemic total synthesis of dactyloidin and demethyldactyloidin through the DL -proline-catalyzed Knoevenagel condensation/[4 + 2] cycloaddition cascade. *Org. Biomol. Chem.* **13**, 9977–9983 (2015).

3. F. Chen, *et al.*, Remote Migratory Cross-Electrophile Coupling and Olefin Hydroarylation Reactions Enabled by in Situ Generation of NiH. *J. Am. Chem. Soc.* **139**, 13929–13935 (2017).

4. A. C. Bissember, A. Levina, G. C. Fu, A Mild, Palladium-Catalyzed Method for the Dehydrohalogenation of Alkyl Bromides: Synthetic and Mechanistic Studies. *J. Am. Chem. Soc.* **134**, 14232–14237 (2012).

5. Y. Liu, J. Cornella, R. Martin, Ni-Catalyzed Carboxylation of Unactivated Primary Alkyl Bromides and Sulfonates with $CO_2$. *J. Am. Chem. Soc.* **136**, 11212–11215 (2014).

6. M. Thompson, C. Carkner, N. J. Mosey, N. Kapernaum, R. P. Lemieux, Tuning the mesomorphic properties of phenoxy-terminated smectic liquid crystals: the effect of fluoro substitution. *Soft Matter* **11**, 3860–3868 (2015).

7. G.-Z. Wang, R. Shang, W.-M. Cheng, Y. Fu, Irradiation-Induced Heck Reaction of Unactivated Alkyl Halides at Room Temperature. *J. Am. Chem. Soc.* **139**, 18307–18312 (2017).

8. M. R. E. S. Aly, H. A. Saad, S. H. Abdel-Hafez, Synthesis, antimicrobial and cytotoxicity evaluation of new cholesterol congeners. *Beilstein J. Org. Chem.* **11**, 1922–1932 (2015).

9. J. L. Romera, J. M. Cid, A. A. Trabanco, Potassium iodide catalysed monoalkylation of anilines under microwave irradiation. *Tetrahedron Lett.* **45**, 8797–8800 (2004).

10. G. Lai, P.-Z. Tan, P. Ghoshal, A One-Pot Method for the Efficient Conversion of Aryl- and Acyl-substituted Methyl Alcohols into Chlorides. *Synth. Commun.* **33**, 1727–1732 (2003).

11. M. Paramanik, R. Singh, S. Mukhopadhyay, S. K. Ghosh, Catalytic nucleophilic fluorination by an imidazolium ionic liquid possessing trialkylphosphine oxide functionality. *J. Fluor. Chem.* **178**, 47–55 (2015).

12. M. H. Ngai, *et al.*, Click-based synthesis and proteomic profiling of lipstatin analogues. *Chem. Commun.* **46**, 8335 (2010).

13. K. Matsumoto, S. Fujie, K. Ueoka, S. Suga, J. Yoshida, An Electroinitiated Cation Chain Reaction: Intramolecular Carbon–Carbon Bond Formation between Thioacetal and Olefin Groups. *Angew. Chem. Int. Ed.* **47**, 2506–2508 (2008).

14. H. T. Dao, U. Schneider, S. Kobayashi, Indium( I )-catalyzed alkyl–allyl coupling between ethers and an allylborane. *Chem Commun* **47**, 692–694 (2011).

15. T. Yang, L. Lu, Q. Shen, Iron-mediated Markovnikov-selective hydro-trifluoromethylthiolation of unactivated alkenes. *Chem. Commun.* **51**, 5479–5481 (2015).

16. X. Wu, L. Chu, F.-L. Qing, Silver-Catalyzed Hydrotrifluoromethylation of Unactivated Alkenes with $CF_3SiMe_3$. *Angew. Chem.* **125**, 2254–2258 (2013).

17. F. Wang, P. Xu, F. Cong, P. Tang, Silver-mediated oxidative functionalization of alkylsilanes. *Chem. Sci.* **9**, 8836–8841 (2018).

18. G. Z. Zheng, T. H. Chan, Regiocontrolled Hydrosilation of .alpha.,.beta.-Unsaturated Carbonyl Compounds Catalyzed by Hydridotetrakis(triphenylphosphine)rhodium(I). *Organometallics* **14**, 70–79 (1995).

19. M. Tobisu, H. Nakai, N. Chatani, Platinum and Ruthenium Chloride-Catalyzed Cycloisomerization of 1-Alkyl-2-ethynylbenzenes: Interception of π-Activated Alkynes with a Benzylic C−H Bond. *J. Org. Chem.* **74**, 5471–5475 (2009).

20. M. Movassaghi, O. K. Ahmad, A Stereospecific Palladium-Catalyzed Route to Monoalkyl Diazenes for Mild Allylic Reduction. *Angew. Chem. Int. Ed.* **47**, 8909–8912 (2008).

21. M. Movassaghi, O. K. Ahmad, A Stereospecific Palladium-Catalyzed Route to Monoalkyl Diazenes for Mild Allylic Reduction. *Angew. Chem. Int. Ed.* **47**, 8909–8912 (2008).

22. Y.-L. Lai, J.-M. Huang, Palladium-Catalyzed Electrochemical Allylic Alkylation between Alkyl and Allylic Halides in Aqueous Solution. *Org. Lett.* **19**, 2022–2025 (2017).

23. A. R. Katritzky, A. M. El-Mowafy, Pyrylium-mediated conversion of primary alkyl primary amines into olefins via tetrahydrobenzoacridiniums: a mild alternative to the Hofmann elimination. *J. Org. Chem.* **47**, 3506–3511 (1982).

24. C. Mao, S. Zhou, C. Qian, J. Ruan, X. Chen, Efficient Dehydration of $C_{6–10}$ -α,ω-Alkanediols to Alkadienes as Catalyzed by Aliphatic Acids. *ACS Omega* **4**, 17588–17592 (2019).
